# C1q/tumor necrosis factor-related protein-3-engineered mesenchymal stromal cells attenuate cardiac impairment in mice with myocardial infarction

**DOI:** 10.1038/s41419-019-1760-5

**Published:** 2019-07-11

**Authors:** Zhengbin Zhang, Liwen Zhu, Pan Feng, Yanzhen Tan, Bing Zhang, Erhe Gao, Xiaowu Wang, Chongxi Fan, Xiaoming Wang, Wei Yi, Yang Sun

**Affiliations:** 1 Department of Geriatric, Xijing Hospital, the Fourth Military Medical University, 710032 Xi’an, China; 2 Department of Cardiovascular Surgery, Xijing Hospital, the Fourth Military Medical University, 710032 Xi’an, China; 3 Department of Cardiology, The First Affiliated Hospital of Xi’an Medical University, 710077 Xi’an, China; 4 Center for Translational Medicine, Lewis Katz School of Medicine at Temple University, 19140 Philadelphia, PA USA; 5 Department of Biomedical Engineering, the Fourth Military Medical University, 710032 Xían, China

**Keywords:** Cell delivery, Mesenchymal stem cells, Myocardial infarction

## Abstract

Mesenchymal stromal cells (MSCs) transplantation offers an attractive alternative in myocardial infarctive therapy. However, poor cell engraftment and survival limit their restorative capacity. C1q/tumor necrosis factor-related protein-3 (CTRP3) inhibits reverse remodeling after myocardial infarction (MI) and was found to be secreted by MSCs in our preliminary experiments. We examined whether the overexpression of CTRP3 improved the survival of transplanted MSCs and augmented their efficacy on MI and whether silencing CTRP3 attenuated these effects. For gain-of-function analysis, MSCs overexpressing CTRP3 (LvC3-MSCs), control virus-transfected MSCs (LvNull-MSCs), MSCs alone, or phosphate-buffered saline (PBS) were injected into the peripheral areas of the infarction immediately after coronary artery ligation. For loss-of-function analysis, mice subjected to MI were randomized into groups and administered CTRP3-knockdown MSCs (LvshC3-MSCs), Lvshctrl-MSCs, MSCs, or PBS. Survival rates, cardiac function, and myocardial remodeling in mice were evaluated after 4 weeks. Injection of MSCs or LvNull-MSCs improved the left ventricular ejection fraction, inhibited cardiac fibrosis, and regulated cellular profiles of the infarction border zone 4 weeks after MI compared with those in the PBS group. Furthermore, overexpression of hCTRP3 promoted the efficacy of MSCs in the treatment of MI. However, knocking down CTRP3 impaired that. Coculture experiments confirmed that hCTRP3-enriched conditioned medium (CM) promoted MSCs migration and protected against H_2_O_2_-induced cell damage. Conversely, CM from C3^−/−^ MSCs (CTRP3 knock out) significantly reduced the migration and antioxidative effects of MSCs. CTRP3 protein alone promoted MSCs proliferation and migration by upregulating matrix metalloproteinase 9 (MMP9) and protecting against oxidation by increasing superoxide dismutase 2 (SOD2) and metallothionein 1/2 (MT1/2) expression; and these effects were blocked by pretreatment with the extracellular signal-regulated kinase (ERK1/2) inhibitor U0126. Overexpression of CTRP3 significantly improved the MSCs-based efficacy on MI by increasing cell survival and retention via a mechanism involving ERK1/2-MMP9 and ERK1/2-SOD2/MT1/2 signaling.

## Introduction

Cardiovascular diseases seriously affect health due to high morbidity and mortality rates. Ischemic heart disease remains a leading cause of death worldwide. With the development of drug treatments, interventional therapies, surgery, and other vascular recanalization and cardiac-assist technologies, the mortality rates of patients with acute myocardial infarction (MI) have decreased significantly. However, adverse remodeling secondary to MI has poor prognosis, with irreversible evolution toward ultimate heart failure. Currently, the number of patients who die of adverse remodeling far exceeds the number of those who die of acute MI^1^. Hence, it is necessary to explore how to prevent adverse remodeling after MI.

Mesenchymal stromal cells (MSCs) are lead candidates for cellular therapy for heart diseases because of their several advantages, including ease of isolation and expansion, paracrine effects and applications in allogeneic “off-the-shelf” transplantation. Many preclinical studies have confirmed their safety and restorative effects. Nonetheless, the results of clinical studies have not been remarkable, and the application of MSCs has been limited because of poor survival and engraftment in the ischemic environment, a prerequisite for cell therapy^2^. Under ischemic conditions, the decrease in antioxidants and the increase in oxidative stress results in elevated levels of damaging ROS in the infarcted heart^3^. Hypoxia and oxidative stress generated in the infarct zone have been hypothesized to contribute to transplanted cell death^4^. As such, multiple approaches have been used to improve cell survival and function by inhibiting oxidative stress-induced apoptosis and increasing the tolerance of injected cells to ischemia and hypoxia^5–8^. Of them, genetic modification of donor cells may be a promising option^5,6^.

C1q/TNF-related proteins (CTRPs) are a newly discovered adipokine family with 15 members. Addition of exogenous CTRP3 has protective effects on pathological remodeling after MI by inhibiting cardiomyocyte apoptosis and myofibroblast differentiation and indirectly increasing angiogenesis^9,10^. Downregulated CTRP9 (homologous to CTRP3) in the heart impairs therapeutic ability of transplanted cells^11^. In our preliminary experiment, we found that MSCs from mouse bone marrow expressed and secreted CTRP3. Based on the above theories and new findings, we hypothesize: genetic manipulation targeting CTRP3 in MSCs could benefit MSCs and enhance MSCs-based therapy in MI. Hence, in this study, we aimed to determine whether overexpression of CTRP3 in MSCs improved their curative effects on MI, investigate the effects of overexpressing CTRP3 on MSCs survival and retention in infarcted hearts, and elucidate the underlying mechanisms.

## Materials and methods

All experiments were approved by the Fourth Military Medical University Committee on Animal Care. Eight-week-old C57BL/6J male mice were provided by the Experimental Animal Center of the Fourth Military Medical University (Xi’an, China). CTRP3-knockout (KO) C57BL/6J mice were supplied by K&D Gene Technology Co., Ltd. (Wuhan, China). U0126 was purchased from MCE (HY-12031)

### Isolation, culture, and characterization of MSCs

MSCs were isolated from mouse bone marrow aspirates using standard protocols^12^. An aspirate of bone marrow was harvested by flushing the tibia and femoral marrow compartments under sterile conditions, and aspirates were then cultured in C57BL/6 Mouse MSC Basal Medium (cat. no. MUBMX-90011; Cyagen Biosciences, Guangzhou, China), supplemented with 10% fetal bovine serum at 37 °C in an incubator containing 5% CO_2_. MSCs was isolated from the whole aspirate through their preferential attachment to tissue culture plastic on the basis of frequent medium changes, and adherent marrow cells were subcultured to passage 4 (P4). To validate the purified population of MSCs, flow cytometry was used to characterize surface markers. P4 MSCs were detached from plates using 0.25% trypsin solution containing 0.01% ethylenediaminetetraacetic acid and resuspended in Flow Cytometry Stain (cat. no. 4311084; ThermoFisher) at a density of 1 × 10^7^ cells/mL. Nonspecific Fc receptor was blocked with anti-CD16/32 antibodies (cat. no. 14-0161; eBioscience), and the cells were then incubated for 30 min on ice away from light with the following antibodies: anti-CD29-fluorescein isothiocyanate (FITC; cat. no. 11-0291; eBioscience), anti-CD34-FITC (cat. no. CBL555F; Millipore), anti-CD44-phycoerythrin (PE; cat. no. 103007; Biolegend), and anti-CD45-FITC (cat. no. 11-0461; eBioscience). Stained cells were resuspended in flow cytometry staining buffer (cat. no. 00-4222-26; eBioscience) for flow cytometry analysis. Osteogenic and adipogenic differentiation of MSCs were assessed by Alizarin Red S and Oil Red O staining (cat. nos. MUBMX-90021 and MUBMX-90031, respectively; Cyagen Biosciences) according to the manufacturer’s protocol.

### Establishment of cell stress models

Two stress models were established to mimic the harsh environment in the ischemic heart: (1) serum deprivation and hypoxia culture; (2) serum deprivation culture with H_2_O_2_^8^.

MSCs were cultured in normal culture medium to 80% confluence and then normal culture medium was replaced by serum-free medium. After overnight, H_2_O_2_ was added to serum-free medium (H_2_O_2_ final concentration, 400 μM) for another 24 h incubation to establish cell serum deprivation and H_2_O_2_ stress model.

Hypoxia and serum deprivation model in vitro was established as described previously^13^. Normal culture medium was replaced by Hanks buffer (Gibco, 14175079). MSCs were placed in a Napco 8000 WJ hypoxia (1% O_2_, 5% CO_2_, 94% N_2_) incubator (Thermo Fisher Scientific, Inc.) at 37 ℃ for 12 h. MSCs were cultured in a normal CO_2_ incubator. (95% air, 5% CO_2_) as the control group.

### Lentivirus vectors and MSCs transduction

Green fluorescent protein (GFP)-conjugated *hCTRP3* lentivirus (LvCTRP3) and control vector (LvNull) expressing GFP alone were constructed simultaneously (Shanghai Genechem Co., Ltd.). In addition, for knockdown of CTRP3, CTRP3-specific shRNA (5′ to 3′ sequence: GCCATGGAGATTATGGATTTC) was package into lentivirus (LvshC3) from Genepharma Inc. (Shanghai, China), and the Lv3-NC vector (5′ to 3′ sequence: TTCTCCGAACGTGTCACGT) served as a control (Lvshctrl). The maps of two types of lentiviral vectors were respectively in Figs. S1a and S3a. MSCs transduction was performed according to the manufacturer’s instructions. Briefly, P4 MSCs were cultured in C57BL/6 Mouse MSC Complete Medium to 40–50% confluence and then cultured with C57BL/6 Mouse MSC Basal Medium overnight. The cells were infected at a multiplicity of infection of 50 for 48 h in the presence of 5 μg/mL polybrene. Transduction efficiency was assessed by flow cytometry.

### Animal model of MI, MSCs transplantation, and study protocol

The mouse model of permanent MI has been described previously^14^. Briefly, mice were anesthetized with 2% isoflurane. The heart was exposed by left thoracotomy after fixation, and the left anterior descending branch of the coronary artery was then ligated with 6-0 silk sutures. Anterior wall blanching suggested that ligation of the coronary artery was a success. Next, 2 × 10^5^ MSCs suspended in 25 μLphosphate-buffered saline (PBS) or 25 μL PBS alone was injected intramyocardially into the infarct border zone in three different areas immediately after MI. When investigating the effects of MSCs overexpressing CTRP3 on MI, mice were randomized into the following groups: (1) MI + PBS (*n* = 20); (2) MI + MSCs (*n* = 22); (3) MI + LvC3-MSCs (*n* = 26); and (4) MI + LvNull-MSCs (*n* = 19). When investigating the effects of CTRP3-knockdown MSCs on MI, mice were grouped as follows: (1) MI + PBS (*n* = 20); (2) MI + MSCs (*n* = 20); (3) MI + LvshC3-MSCs (*n* = 20); and (4) MI + Lvshctrl-MSCs (*n* = 20). Survival was recorded for 28 days, and echocardiographic parameters were obtained on days 1, 7, and 28 after MI.

### Echocardiographic analysis

The cardiac function of all mice was assessed by transthoracic echocardiograph using a VisualSonics VeVo 2100 Imaging System. Left ventricular end diastolic diameter and end systolic diameter from M-mode were recorded, and left ventricular ejection fraction (LVEF) was calculated.

### Masson staining

The animals were sacrificed at 4 weeks after MI, and hearts were fixed with 4% paraformaldehyde overnight and embedded in paraffin. Subsequently, the hearts were coronally sectioned to 5 μm thickness. Five sections per heart were stained with Masson’s trichrome. Fibrosis was measured by ImageJ software and determined as the percentage of the total area of the left ventricle.

### Terminal deoxynucleotidyl transferase dUTP nick end labeling (TUNEL) staining

Tissue apoptosis was detected 4 weeks after MI. Three to five sections from each heart were stained using a TUNEL kit (cat. no. 11684817910; Roche Diagnostics Corporation) according to the manufacturer’s instructions. Quantitative analysis was performed by counting the number of apoptotic cells from four to six fields per slide. For cultured cells in vitro, apoptosis was quantified using a TUNEL kit as described above. At least 100 cells/sample were randomly selected and counted to calculate the percentage of apoptotic cells.

### Immunofluorescence staining

Frozen sections of heart tissues were prepared by fixing with OCT (cat. no. 4583; Sakura Finetek USA, Inc.) and sectioned at 4 weeks after MI. Slides were permeabilized by 0.1% Triton for 10 min, blocked in 1% bovine serum albumin, and then incubated with following primary antibodies: anti-Von Willebrand factor (vWF; cat. no. ab6994; Abcam, Cambridge, UK), anti-α-smooth muscle actin (SMA; cat. no. ab5694; Abcam), and anti-vimentin (cat. no. ab8978; Abcam). After rinsing with PBS, the cells were incubated in PE-conjugated goat anti-mouse IgG secondary antibodies (cat. no. A10703G-PE; Solarbio) or Cy3-conjugated goat anti-rabbit IgG (cat. no. EK022; Zhuangzhibio, China). Nuclei were stained with 4′,6-diamidino-2-phenylindole (DAPI).

### Monitoring of the survival and retention

Eight-micron-thick serial frozen sections near the ligation suture were prepared on days 1, 7, and 14 after MI, as described above for immunofluorescence staining. The slides were scanned by lasers were scanned laser scanning confocal microscopy, and digital images were analyzed using Image-Pro Plus. MSCs survival and retention rates were expressed as the ratio of the GFP-positive cells (MSCs) per 1000 nuclei. To investigate MSCs differentiation, frozen slides of tissues from day 7 after MI were incubated with primary antibodies against vWF (red), αSMA (red), and then incubated with PE-conjugated goat anti-mouse IgG secondary antibodies (cat. no. A10703G-PE; Solarbio) as described above. Nuclei were stained with DAPI. The percentages of vWF- or αSMA- GFP double-positive cells represented the numbers of differentiated vascular endothelial cells (ECs) and vascular smooth muscle cells (SMCs), respectively. All data were analyzed by independent blinded researchers.

### Preparation of recombinant hCTRP3 protein

Construction and expression of hCTRP3 were performed as previously reported^9^. Briefly, the *hCTRP3* gene was generated by GENEWIZ (Jiangsu, China) and cloned into the prokaryotic protein expression vector pET30a (Novagen, Merck, USA). The hCTRP3 prokaryotic expression vector was transferred into a BL21 (DE3) bacterial protein expression host. BL21 (DE3)/pET30a-hCTRP3 was grown in LB medium and shaken at 37 °C for 3 h. isopropyl-B d-thiogalactoside was added to the medium (final concentration: 1 mM). The solution was shaken for 4–5 h at 16 °C and subjected to centrifugation at 5000 rpm. Proteins were purified under native conditions by HisTrap HP per the manufacturer’s instructions (No. 17-5247-01; GE Healthcare, USA). Endotoxin was removed using an endotoxin-removing column (Pierce High Capacity Endotoxin Removal Spin Columns; Pierce, ThermoFisher, USA). Proteins were desalted and concentrated by centrifugation (cat. no. UFC900396; Millipore).

### Cell growth assay

Cell growth assays were performed using a Cell Counting Kit-8 (CCK-8; Beyotime Institute of Biotechnology, Nanjing, China) based on the manufacturer’s protocol. Cells were incubated with 10% CCK-8 solution in 96-well plates for 2–4 h at 37 °C. The absorbance was detected at 450 nm using a microplate spectrophotometer.

### Two-chamber migration assay

To measure cell migration, 2 × 10^5^ MSCs were plated in transwell inserts (8 μm pore size; cat. no. 3422; Corning Inc., NY, USA) with the upper surface coated using basement membrane extract (cat. no. 3455-096-02; Trevigen). Receiver wells in 24 wells were seeded with MSCs, LvC3-MSCs, LvNull-MSCs, or C3^−/−^ MSCs. For CTPP3 direct simulation assays, receiver wells were set up with 600 μL serum-free medium containing different concentrations of hCTRP3. The inserts were then placed into 24-well plates, and cultures were incubated for 24 h. Cells on the upper surface were removed, and cells at the bottom of the transwell membrane were fixed and stained with 0.1% Crystal Violet Solution (diluted with methanol). The number of migrated cells per membrane was counted by a single blinded researcher via microscopy.

### Flow cytometry analysis for cell apoptosis assays

Flow cytometry was used to assess cell apoptosis with an Annexin V-FITC/PI staining kit (cat. no. A005-3; 7sea Biotech, China) following the manufacturer’s instructions. Briefly, MSCs were treated, collected, washed with ice-cold PBS, and counted. In total, 5 × 10^4^ cells were incubated in 195 μL FITC-conjugated annexin V binding buffer containing 5 μL annexin V-FITC and 10 μL propidium iodide for 20 min at room temperature in the dark. Apoptotic cells were quantified using a flow cytometer.

### Lactate dehydrogenase (LDH) assay

H_2_O_2_-induced cell death was evaluated by LDH release assay. MSCs were exposed to 400 μM H_2_O_2_ (diluted with serum-free medium) for 24 h. The culture supernatants were collected and detected using an LDH cytotoxicity assay kit (cat. no. A020-2; Beyotime, China), according to the manufacturer’s instructions. The absorbance of all samples was read at 490 nm using a SpectraMax M5 microplate reader (Molecular Devices, Eugene, OR, USA).

### Quantitative polymerase chain reaction (qPCR)

At 24 h after treatment, total RNA was extracted from cultured cells with an RNA simple Total RNA Kit (cat. no. DP419; TIANGEN), according to the manufacturer’s protocol. cDNA synthesis was carried out using Prime Script RT Master Mix (cat. no. RR036A; Takara, Shiga, Japan) following the manufacturer’s instructions. Real-time PCR was performed with a CFX-96 Content Real-time System using TaKaRa SYBR Premix Ex Taq II (cat. no. RR820A; Takara). The PCR protocol was as follows: 95 °C for 30 s, 1 cycle; 95 °C for 5 s, 60 °C for 30 s, 72 °C for 40 s, 40 cycles. GAPDH or β-actin served as a qPCR amplification endogenous control. The PCR primers were purchased from GenScript Biotech Corp. (Nanjing, China), and primer sequences are listed in Table S1. Heml Heatmap Illustrator was used to display the expression patterns as a heat map.

### In-gel zymography for assessing matrix metalloproteinase (MMP) activity

MMPs activity was determined by in-gel zymography, as described previously^15^. Briefly, MSCs in the logarithmic phase were treated in 6-well plates for 24 h. CM was collected and centrifuged at 4 ℃ and 2000 rpm for 10 min. The protein concentrations of samples were determined by the BCA method. The sample was mixed with 2 × hsodium dodecyl sulfate (SDS)-polyacrylamide gel electrophoresis non-reducing buffer (100 mM Tris-HCl, 4% SDS, pH 6.8, 20% glycerol, 0.02% Bromophenol blue), and the loading volume was adjusted to ensure the same protein quantity. The zymographic gel was made using a modified method, with a conventionally prepared 5% stacking gel and 8% separating gel, supplemented with substrate (1% Substrate G). After electrophoresis, the gel was washed with washing buffer (25 mL Triton X-100 and 975 mL sterile diH_2_O) and activated through incubation with zymographic development buffer (50 mM Tris [pH 7.4], 10 mM CaCl_2_, 0.02% NaN_3_, sterile diH_2_O). The gel was stained with Coomassie blue solution for 1 h at room temperature (0.125 g Coomassie brilliant blue R-250, 1 mL acetic acid, 45 mL ethanol, and 54 mL sterile diH_2_O) and decolored in destaining solution I (50 mL acetic acid, 125 mL ethanol, and 325 mL sterile diH_2_O) and solution o (37.5 mL acetic acid, 25 mL ethanol, and 437.5 mL diH_2_O) until clear bands were observed. Gel scanning and quantification were carried out with a Bio-Rad Imaging System.

### Western blotting

Cells supernatant was collected and concentrated as described previously^16^. The culture medium was washed and replaced by serum-free medium. After another 24 h, CM was collected and cells were counted for normalization: we used medium generated by 5 × 10^6^ MSCs. After removing cell debris, CM was concentrated to 500 μL by Amicon® Ultra-15 3 K Devices (Millipore, MA, USA.) per manufacturer’s introduction. 30 μL CM sample was loaded for electrophoresis. Tissue and cell samples were harvested and lysed, after which the protein concentration was determined using a BCA Protein Assay Kit (cat. no. 23227; Thermo Fisher Scientific, Inc.) according to the manufacturer’s protocol. Proteins were subjected to electrophoresis and transferred to polyvinylidene fluoride membranes. The membranes were incubated with 5% nonfat dry milk in TBST for 2 h at room temperature and subsequently incubated overnight at 4 °C with primary antibodies as follows: anti-CTRP3 (cat. no. ab36870; Abcam), anti-CCNA2 (cat. no. ab38; Abcam), anti-CCNB1 (cat. no. 4135; Cell Signaling Technology, Danvers, MA, USA), anti-Nusap1 (cat. no. PA5-42841; Invitrogen, Carlsbad, CA, USA), anti-MMP3 (cat. no. sc-21732; Santa Cruz Biotechnology, Santa Cruz, CA, USA), anti-MMP9 (cat. no. ab38898; Abcam), anti-MT1/2 (cat. no. ab12228; Abcam), anti-SOD2 (cat. no. 13141; Cell Signaling Technology), anti-phospho-AKT (cat. no. 4058; Cell Signaling Technology), anti-AKT (cat. no. 2967; Cell Signaling Technology), anti-phospho-extracellular signal-regulated kinase (ERK1/2; cat. no. 9106S; Cell Signaling Technology), anti-ERK1/2 (cat. no. 4696S; Cell Signaling Technology), anti-phospho-AMP-activated protein kinase (AMPK; cat. no. 2531; Cell Signaling Technology), anti-AMPK (cat. no. 2532; Cell Signaling Technology), anti-phospho-c-Jun N-terminal kinase (JNK; cat. no. EAP1789; Elabscience), anti-JNK (cat. no. ab199380; Abcam), anti-β-tubulin (cat. no. AT0003; CMCTAG), anti-β-actin (cat. no. AT0001; CMCTAG), and anti-GAPDH (cat. no. AT0002; CMCTAG). After washing with TBST, the blots were incubated for 2 h with anti-rabbit or anti-mouse secondary antibodies (1:1,000; cat. nos. ZB-2301 and ZB-2305; Zhongshan Company, China) at room temperature. The blots were visualized and analyzed using a Bio-Rad Imaging System (Hercules, CA, USA).

### Statistical analysis

All data in the text and figures are presented as means ± SEMs unless otherwise specified. The data were analyzed using GraphPad Prism-6 statistic software (La Jolla, CA, USA). Differences between two groups were analyzed by unpaired, two-tailed t-tests, and one-way analysis of variance (ANOVA) followed by Bonferroni post-hoc tests was used to compare between three or more groups. Survival data analysis was conducted via the Kaplan-Meier method followed by the log-rank test. Time and group differences were determined by two-way ANOVA followed by post-hoc tests. Differences with *P* values of less than 0.05 were considered statistically significant.

## Results

### Cells isolated from mouse bone marrow showed MSCs characteristics, and serum deprivation plus H_2_O_2_ treatment downregulated CTRP3 expression and secretion in MSCs

**Fig. 1:**
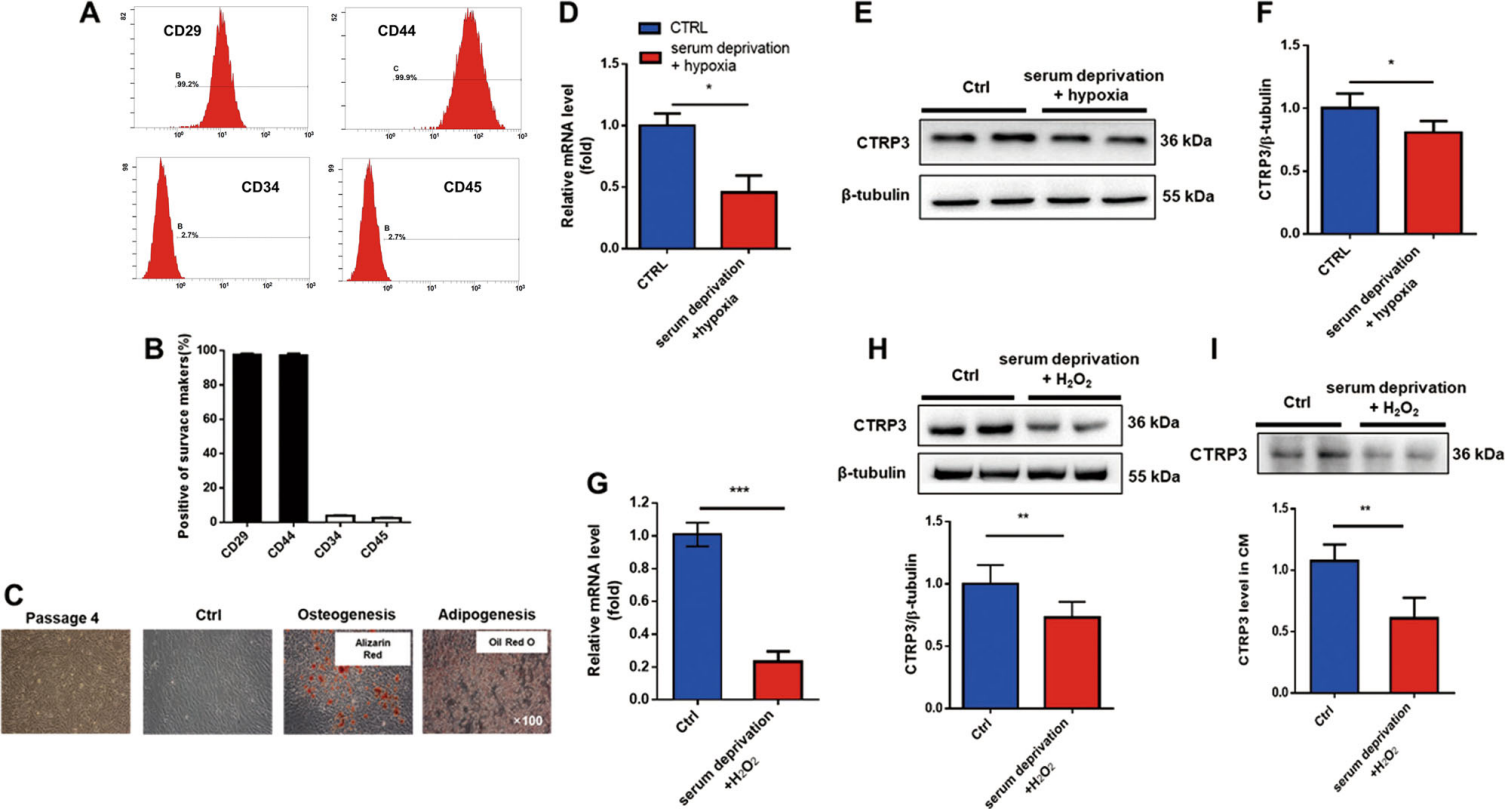
Serum deprivation and oxidative injury inhibited the expression and secretion of CTRP3 in MSCs. **a**, **b** Surface marker molecules expressed in passage 4 MSCs were analyzed by flow cytometry and quantified by image analysis of positive cells. Data are means ± SEMs (*n* = 4). **c** Representative image of MSCs morphology (passage 4) and multilineage differentiation capacity into osteoblasts and adipocytes, Images were at original magnification, ×100. **d** RT-PCR was used to evaluate CTRP3 mRNA levels in MSCs exposed to serum deprivation and hypoxia (*n* = 4). **e**, **f** Western blotting and quantification of CTRP3 in MSCs (*n* = 6). **g** RT-PCR analysis for MSCs treated with serum deprivation plus 400 μM H_2_O_2_ for 24 h (*n* = 4). **h** Western blotting and quantification of CTRP3 in MSCs cell lysates (*n* = 6). **i** Western blots and quantification of CTRP3 in conditioned medium (CM) from MSCs treated with serum deprivation plus H_2_O_2_ (*n* = 5). Data are means ± SEMs. *P < 0.05; **P < 0.01; ***P < 0.001 versus the control

MSCs were isolated and expanded from mouse bone marrow. MSCs displayed a homogenous spindle-shaped morphology after four passages (Fig. S1b). Flow cytometry analysis showed that cultured MSCs expressed the MSCs markers CD29 and CD44 and were devoid of the hematopoietic lineage markers CD34 and CD45 (Fig. 1a, b). MSCs pluripotency was demonstrated by evaluating their differentiation capacity into adipogenic and osteogenic lineages induced in vitro (Fig. 1c).

Two in vitro stress models, serum deprivation under hypoxia, and serum deprivation combined with H_2_O_2_, were established to evaluate CTRP3 expression in harsh microenvironment in the ischemic heart. qPCR and western blotting results indicated that CTRP3 expression (for both mRNA and protein levels) were suppressed in MSCs under serum deprivation and hypoxia stimulation (Fig. 1d–f). MSCs also displayed decreased expression of CTRP3 in the setting of serum deprivation combined with H_2_O_2_ (Fig. 1g, h). Moreover, western blotting of CTRP3 in cell supernatant showed that CTRP3 secretion were decreased in MSCs exposed to serum deprivation plus H_2_O_2_ stimulation (Fig. 1i). These results suggested that CTRP3 may play key roles in MSCs functions.

### CTRP3 overexpression enhanced the effects of MSCs on improvement of survival, restoration of cardiac function, and amelioration of remodeling after MI

A new recombinant lentiviral vector expressing both *hCTPR3* gene and reporter *GFP* simultaneously was constructed, and a control lentiviral vector only harboring *GFP* was designed. After transfection of MSCs, robust expression of GFP was observed (Fig. S1b); Flow cytometry analysis showed ~80% infection efficiency on day 3 post-infection, which remained stable on day 7 post-infection (Fig. S1c). Moreover, the proportion of GFP-positive cells did not decrease with cell passage (Fig. S1c), indicating the lentivirus could efficiently and stably infects MSCs. Western blot analysis in MSCs lysates indicated that total CTRP3 levels were higher in the LvC3-MSCs group than that in the LvNull-MSCs (Fig. S1d), demonstrating the successful construction of CTRP3-overexpressing MSCs.

### Survival and cardiac function

**Fig. 2:**
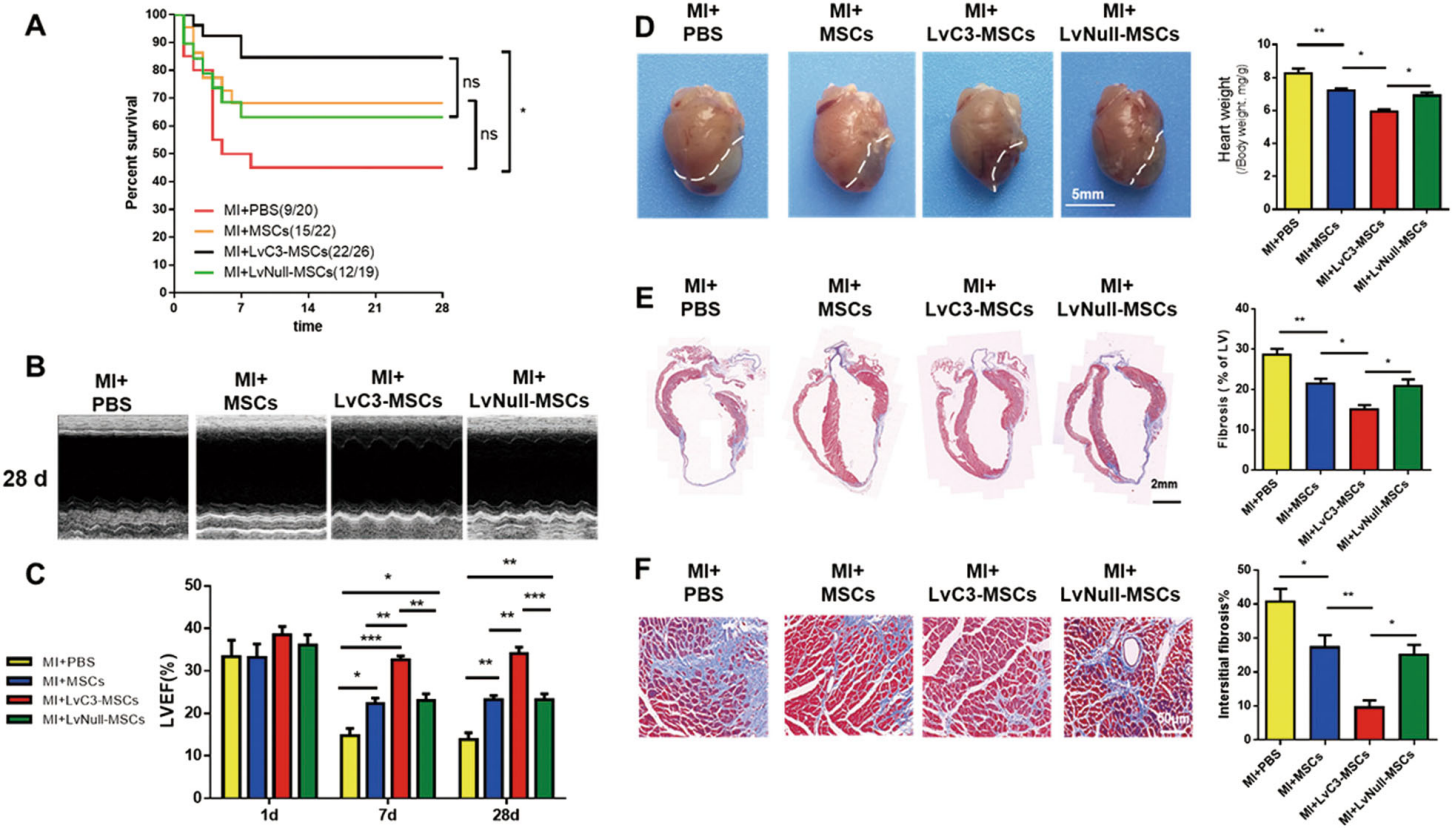
CTRP3-overexpressing MSCs improved survival, restored left ventricular cardiac function, and abated left ventricular cardiac remodeling after MI. **a** The survival proportions of all groups after MI at the indicated time points (*n* = 19–26). **b**, **c** Echocardiographic analysis of left ventricle ejection fraction after MI at 1, 7, and 28 days for all groups (*n* = 5). **d** Gross observations and heart weight to body weight ratio (*n* = 5–7). **e** Representative masson staining for left ventricular fibrosis (*n* = 5). **f** Interstitial fibrosis 28 days after MI (*n* = 5). Data are means ± SEMs. *P < 0.05; **P < 0.01; ***P < 0.001. ns, not significant

To investigate the efficacy of MSCs transplantation in vivo, 2 × 10^5^ CTRP3-MSCs or LvNull-MSCs were transplanted into the infarct border zone via intramyocardial injection. Higher CTRP3 levels were found in the border region 7 days after injection of CTRP3-overexpressing MSCs, as demonstrated by western blotting (Fig. S1e). Then we investigated whether CTRP3 overexpression strengthened the capability of MSCs to improve survival and restore cardiac function in mice subjected to permanent MI. As illustrated in Fig. 2a, approximately 50% of the PBS-injected MI mice survived at 4 weeks. Injection with MSCs or LvNull-MSCs caused an obvious reduction in mortality rates, although there were no significant differences compared with PBS injection alone. However, administration of LvC3-MSCs increased post-MI survival rates up to nearly 85% (Fig. 2a), representing a significant difference compared with the MI + PBS group.

Cardiac function was evaluated with echocardiographic analyses at 1, 7, and 28 days after MI (Fig. 2b, c). All groups show no significant differences in LVEF at 1 day after MI. However, a statistically significant improvement in LVEF was observed in MI + MSCs and MI + LvNull-MSCs groups compared with that in the MI + PBS group at 7 days after MI. Furthermore, administration of LvC3-MSCs significantly improved LVEF compared with injection of LvNull-MSCs at 7 and 28 days. These data demonstrated that CTRP3 overexpression promoted the effects of MSCs on increasing survival and cardiac function.

### Cardiac remodeling and fibrosis

**Fig. 3:**
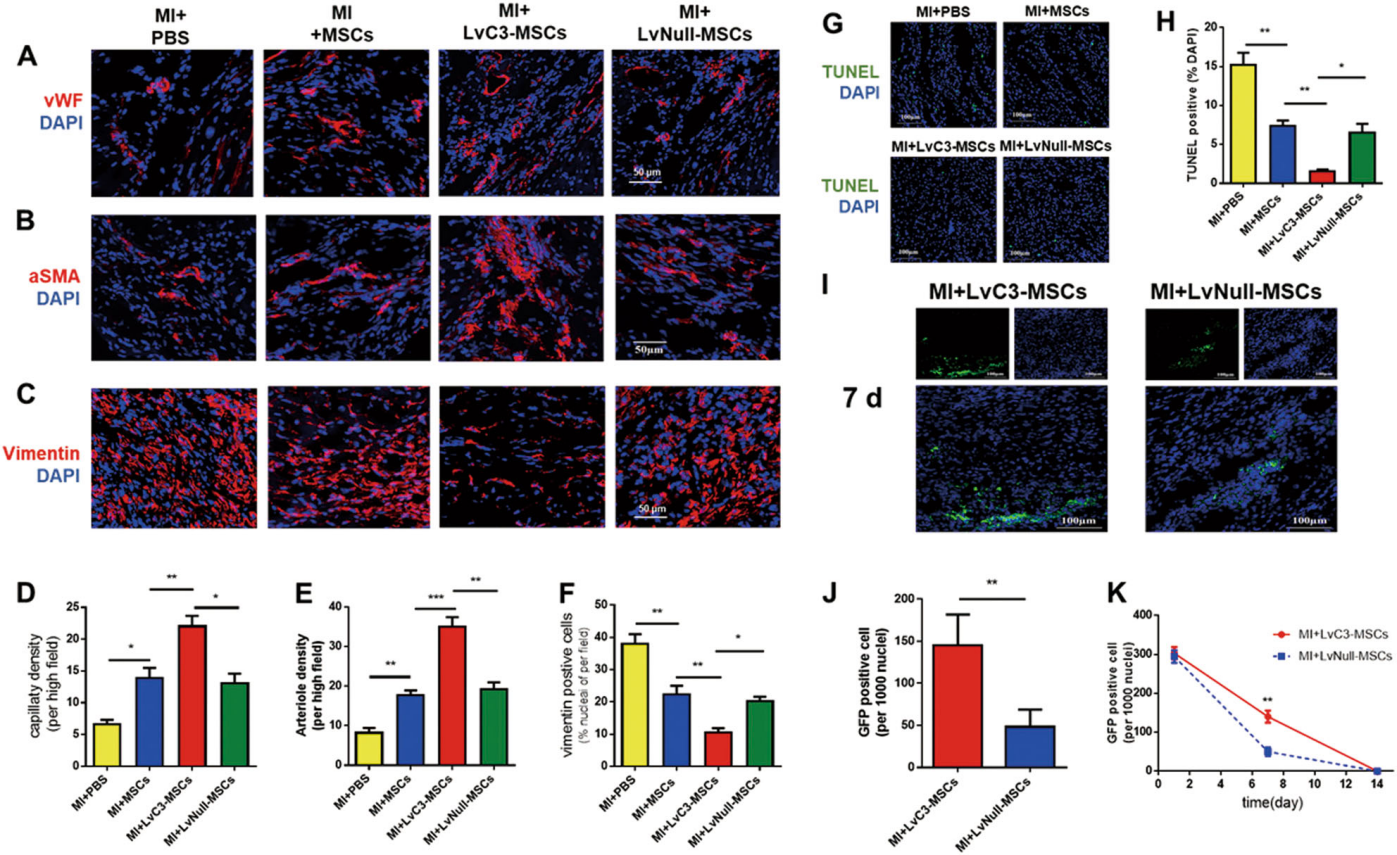
Overexpression of CTRP3 inhibited cardiomyocyte apoptosis, regulated the cellular profile, and enhanced MSCs survival and retention. **a**–**c** Representative confocal microscopic images of vWF (**a**), αSMA (**b**), and vimentin-positive cells (**c**) (red) in the infarct border region 4 weeks after cell injection. Nuclei were stained with DAPI (blue). Quantification of capillary density (**d**), arteriole density (**e**), and fibroblasts (**f**), summarized in bar graphs (*n* = 5). **g**, **h** Representative photographs and quantified analysis of TUNEL-labeled (green) apoptotic cardiomyocytes at 28 days after MI (*n* = 4). **i** Representative images of MSCs in hearts 7 days after MI. **j** Quantification of MSCs in the peri-infarct area, as determined by the ratio of the GFP-positive cells (MSCs) per 1000 nuclei (*n* = 5). **k** Engraftment curves of implanted MSCs were compared at the indicated times (*n* = 5). All data are expressed as means ± SEMs. *P < 0.05; **P < 0.01; ***P < 0.001

Cardiac remodeling was determined by gross anatomy and masson trichrome staining 28 days after MI. As shown in Fig. 2d, compared with the MI + PBS group, both MSCs and LvNull-MSCs reduced heart size and mass, and these changes were more dramatic when LvC3-MSCs were administered. Similarly, the myocyte/fibrotic cell ratio was significantly increased (Fig. 2e), and interstitial fibrosis was visibly attenuated in animals injected with LvC3-MSCs compared with that in animals injected with LvNull-MSCs (Fig. 2f). These findings were supported by the lower fibroblast density in mice receiving LvC3-MSCs, as determined by counting vimentin-positive cells in the peri-infarction zone (Fig. 3c, f).

### Cardiomyocyte apoptosis and cellular profiles in the infarct border zone

To evaluate the effects of LvC3-MSCs treatment on blood vessel density 28 days after MI in vivo, we performed immunofluorescence staining of vWF and αSMA to identify and quantify capillary and small arterioles densities, respectively. As shown in Fig. 3a, b, in the peri-infarcted zone of hearts injected with MSCs or LvNull-MSCs, significant increases in the densities of capillaries and small arterioles were observed compared with that of PBS-treated hearts (Fig. 3d, e). Notably, angiogenesis in LvC3-MSCs-injected hearts increased compared with those in the MI + MSCs and MI + LvNull-MSCs groups (Fig. 3a, b, d, e). In addition, we observed that CTRP3 overexpression via infusion of CTRP3-engineered MSCs dramatically decreased vimentin positive cells (Fig. 3c, f) and enhanced the effects of MSCs on inhibition of myocardial apoptosis in the border zone 28 days after MI (Fig. 3g, h). These results suggested that MSCs played a positive role in regulating cardiac remodeling after MI and that CTRP3 overexpression accelerated the therapeutic effects of MSCs against MI.

### Survival and retention of implanted MSCs were enhanced by CTRP3-transfected MSCs in infarcted hearts

Cell survival and retention were determined by GFP immunostaining analysis of harvested myocardial samples from the injected sites at 1, 7, and 14 days after transplantation. The results showed that GFP-positive MSCs formed a cluster, which decreased over time and survived only for a limited time (Fig. 3i–k). There were no significant differences in the green fluorescent intensity of infarcted hearts between LvC3-MSCs and LvNull-MSCs on day 1, whereas more GFP-positive cells were present in the LvC3-MSCs group 7 days after MI compared with the LvNull-MSCs group (Fig. 3j, k). These data indicated that CTRP3-engineered MSCs enhanced cellular survival and retention. Furthermore, to assess whether CTRP3 overexpression in MSCs enhanced MSCs differentiation in vivo, double immunofluorescence staining was analyzed 7 days after MI. Vascular ECs and vascular smooth muscle cells were labeled with vWF and αSMA respectively. As shown in Fig. S2, confocal microscopic images revealed that only a very small proportion of ECs in the peri-infarct zone were GFP positive, and no significant differences were observed between the LvC3-MSCs and LvNull-MSCs groups for the number of MSCs-derived ECs. Notably, αSMA and GFP double-positive cells were not found. These results suggested that cell differentiation was not the main mechanism responsible for MSCs therapy and that CTRP3 rarely contributed to MSCs differentiation in vivo.

### Silencing CTRP3 significantly abolished the protective effects of MSCs on cardiac function and adverse remodeling

**Fig. 4:**
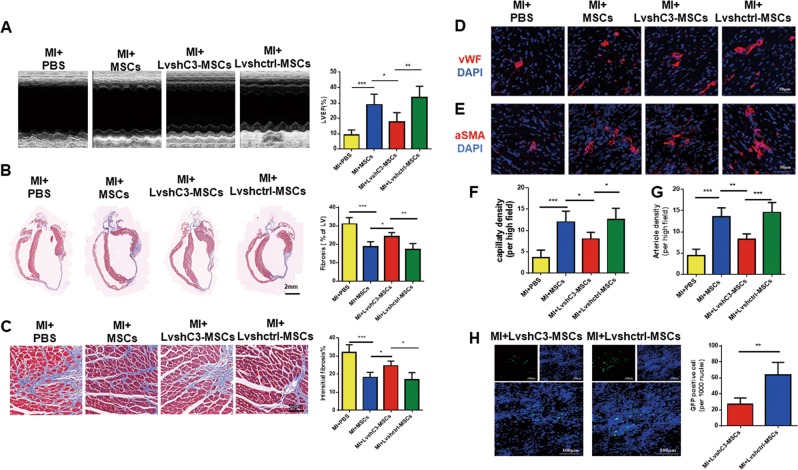
Knockdown of CTRP3 impaired the therapeutic effects of MSCs on cardiac function recovery and cardiac remodeling, related to a decrease in the number of MSCs. **a** Echocardiographic analysis of the left ventricle ejection fraction at 28 days after MI for all groups(*n* = 5). **b** Representative masson staining of left ventricular fibrosis (*n* = 5). **c** Interstitial fibrosis 28 days after MI (*n* = 5). **d**, **e** Representative confocal microscopic images of vWF (**d**) or αSMA-positive cells (**e**) (red) in the infarct border region 4 weeks after MI. Nuclei were stained with DAPI (blue). Quantification of capillary density (**f**) and arteriole density (**g**) (*n* = 5). **h** Representative images of MSCs and quantification in hearts 7 days after MI (*n* = 5). All data were expressed as means ± SEMs. *P < 0.05; **P < 0.01; ***P < 0.001

Because CTRP3-overexpressing MSCs effectively repaired damaged hearts by increasing the survival of transplanted cells, we further determined whether knocking down CTRP3 expression altered MSCs efficacy on MI. LvshC3 infection resulted in significant decrease in CTRP3 levels compared with that in Lvctrl-infected MSCs (Fig. S3c). As illustrated in Fig. 4a, knockdown of CTRP3 attenuated the effects of MSCs on restoring cardiac ejection function. Moreover, compared with the Lvshctrl-MSCs or MSCs groups, LvshC3-MSCs not only significantly increased cardiac fibrosis and interstitial fibrosis (Fig. 4b, c) but also reduced capillary (Fig. 4d, f) and small arteriole densities in the border zone (Fig. 4e, g). Finally, we assessed the survival and retention of transplanted MSCs in each group, and the results showed that silencing CTRP3 reduced the number of engrafted MSCs compared with the Lvshctrl-MSCs group (Fig. 4h). The data suggested that loss of CTRP3 adversely regulated MSCs engraftment and impaired the effects of MSCs on MI.

### CTRP3 secreted by MSCs induced MSCs migration and protected MSCs from H_2_O_2_-induced apoptosis/necrosis

**Fig. 5:**
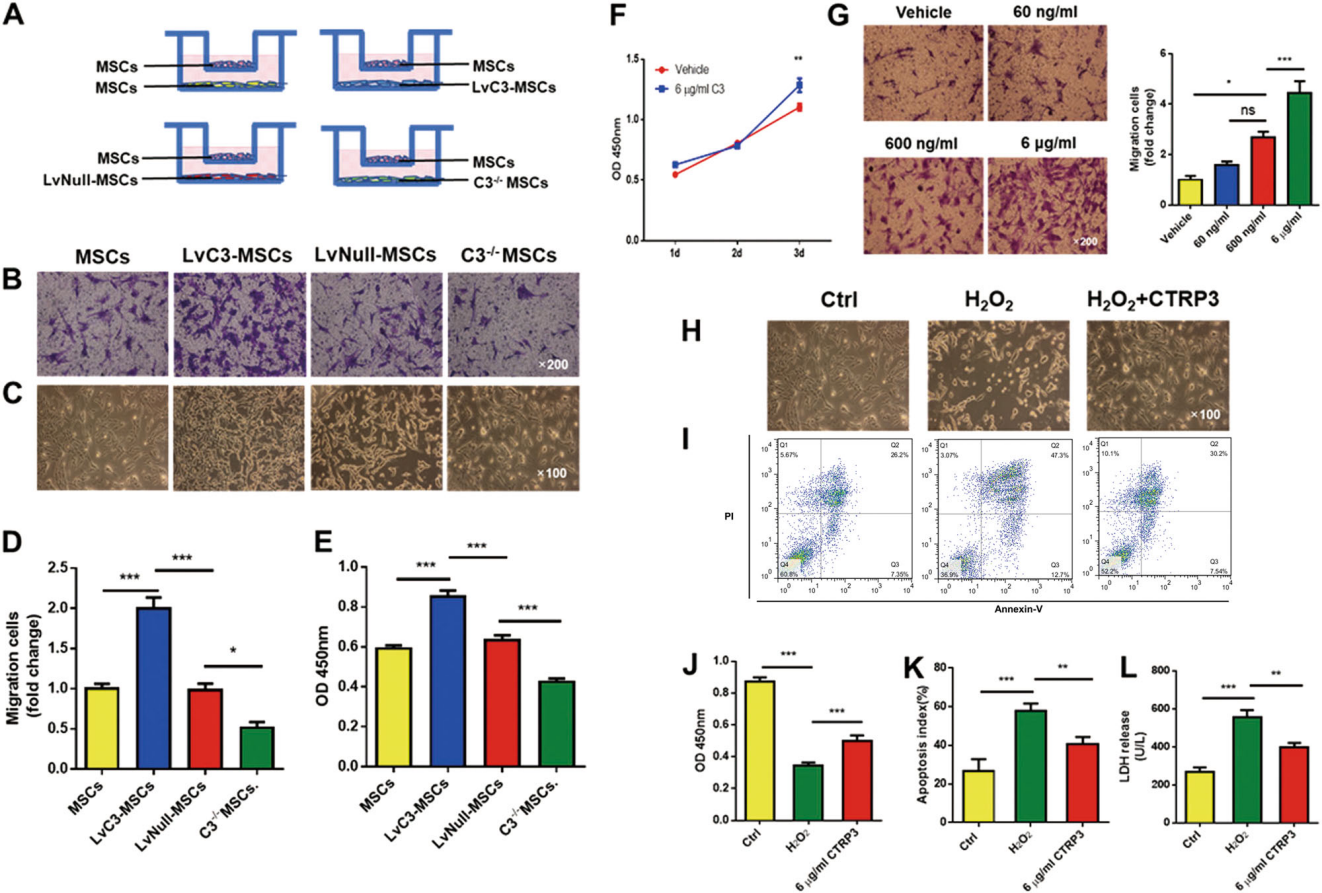
MSCs-derived CTRP3 promoted MSCs proliferation and migration and protected against H_2_O_2_-induced injury. **a** Experimental setting. MSCs were seeded in the upper chambers of trans-well inserts, with MSCs, LvC3-MSCs, LvNull-MSCs, or C3^−/−^ MSCs plated in the lower chambers. **b** and **d** Representative images and bar graph illustrating migration assays for MSCs cocultured with gene-modified MSCs. Normal MSCs were used as a control (*n* = 5). Images were at original magnification, ×200. **c** Bright-field micrographs illustrating MSCs morphology and density when cultured with CMs from gene-modified MSCs and simultaneously exposed to H_2_O_2_ for 24 h. Images of phase contrast were at original magnification, ×100 (**e**) CCK-8 assays were used to measure cell viability for **c** (*n* = 6). **f** Growth curves of MSCs treated with CTRP3 protein alone (6 μg/mL) were measured using CCK-8 assays (*n* = 6–10). **g** The direct effects of CTPR3 on MSCs migration were observed (*n* = 5). Images were at original magnification, ×200. **h** and **j** MSCs survival was measured using CCK-8 assays (*n* = 11). Images were at original magnification, ×100. **i** and **k** Apoptosis was quantified by flow cytometry (*n* = 4). **l** MSCs death was determined by LDH release (*n* = 4). All data are presented as means ± SEMs. *P < 0.05; **P < 0.01; ***P < 0.001. ns, not significant

CTRP3 effectively promotes post-ischemic angiogenesis by enhancing communication between cardiomyocytes and endothelial cells^9^. Our results (Fig. 3a, d) demonstrated that CTRP3 upregulation in MSCs was correlated with increased capillary density in the border zone, consistent with previous reports^9^. In addition, we wondered whether the altered MSCs retention was regulated by CTRP3 secreted by MSCs themselves. Thus, MSCs isolated from CTRP3-KO (knock out) mice were cultured, and loss of protein in CTRP3^−/−^ MSCs CM was confirmed by western blotting (Fig. S4a). Then we evaluated the proliferation and death of MSCs treated with CM from MSCs, LvC3-MSCs, LvNull-MSCs, or C3^−/−^ MSCs. As shown in Fig. S4b, MSCs, LvC3-MSCs, LvNull-MSCs, and C3^−/−^ MSCs were seeded in 6-well dishes and grown to 80% confluence. The culture medium was replaced with serum-free medium. CM was collected after 24 h. To detect cell proliferation ability, MSCs were incubated with CM for 48 h; and to test antioxidant activity, MSCs were incubated with CM and 400 μM H_2_O_2_ for 24 h (Fig. S4b). CCK-8 assays showed that compared with CM from MSCs, neither CTRP3-enriched CM nor CTRP3-deficient CM had pronounced effects on MSCs proliferation at 48 h after treatment (Fig. S4c). Additionally, coculture experiments indicated that MSCs in receiver wells of transwell system with high expression of CTRP3 promoted MSCs migration. In contrast, C3^−/−^ MSCs exhibited reduced migration (Fig. 5a, b, d).

To further determine the effects of oxidative stress, we tested the effects of different CMs on H_2_O_2_-induced MSCs injury. Compared with CM from MSCs or LvNull-MSCs, CM from LvC3-MSCs increased the growth of MSCs exposed to H_2_O_2_, whereas CM from C3^−/−^ MSCs reduced MSCs growth (Fig. 5c, e).

Next, we evaluated the direct effects of CTRP3 protein on the biological behaviors of MSCs. Our data showed that hCTRP3 protein alone stimulated MSCs behaviors, consistent with above experiments. Indeed, hCTRP3 protein promoted MSCs migration in a concentration-dependent manner (Fig. 5g). Additionally, hCTRP3 significantly attenuated MSCs apoptosis and necrosis induced by H_2_O_2_ (Fig. 5h–l). Surprisingly, although experiments using CMs failed to promote MSCs proliferation, direct hCTRP3 treatment for 72 h significantly stimulated MSCs proliferation (Fig. 5f). These findings suggested that CTRP3 secreted by MSCs induced MSCs proliferation and migration and resisted oxidative injury.

### CTRP3 upregulated the cytoprotective proteins SOD2 and MT1/2 and increased MMP9 secretion

**Fig. 6:**
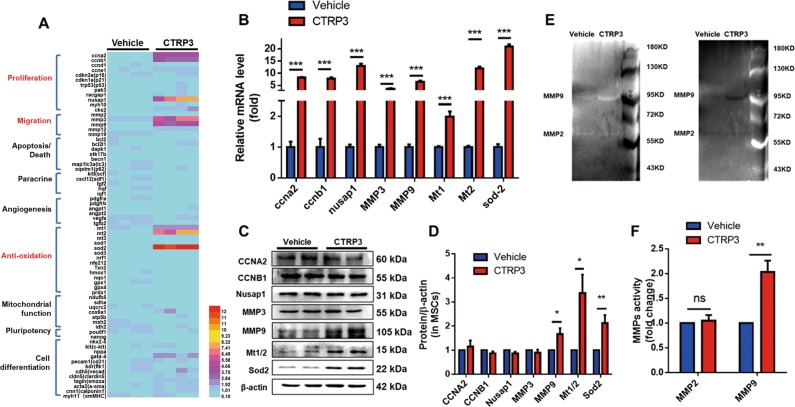
CTRP3 treatment resulted in upregulation of SOD2 and MT1/2 expression levels and increased MMP9 secretion in MSCs. **a** A heat map illustrating the levels of 70 mRNAs related to MSCs biological functions in the CTRP3 and vehicle group (*n* = 4). **b** mRNA and (**c** and **d**) protein expression levels of genes from (**a**) were analyzed by RT-PCR (*n* = 4) and western blotting (*n* = 6–10). The gene and protein expression levels were normalized to that of β-actin. **e**, **f** Representative images of in-gel zymography for analysis of MMPs activity in CM from MSCs (gray and reversed-phase). MMP9 activity was quantified by densitometric analysis (*n* = 4). All data are presented as means ± SEMs. *P < 0.05; **P < 0.01; ***P < 0.001. ns, not significant

To further study the molecular mechanisms, we examined the levels of 70 mRNAs relative to MSCs biological functions^17^. MSCs were cultured after CTRP3 treatment for 48 hours, and mRNA and protein levels were detected. As illustrated in Fig. 6a, b, the following eight genes were significantly upregulated more than 2 folds in MSCs exposed to CTRP3 compared with those in vehicle-treated cells: cyclin A2 (*CCNA2*), cyclin B1 (*CCNB1*), nucleolar and spindle associated protein 1 (*NUSAP1*), *MMP3*, *MMP9, MT1, MT2*, and *SOD2*. Furthermore, western blot analysis confirmed that CTRP3 increased the expression of MMP9 and the antioxidant proteins SOD2 and MT1/2 (Fig. 6c, d). CTRP3 also significantly upregulated MMP9 expression and enhanced MMP9 activity in CM from MSCs, as quantified by western blotting and in-gel zymography (Fig. S5, Fig. 6e, f).

### Activation of ERK1/2 signaling potentiated CTRP3-mediated MSCs proliferation, migration, and cytoprotective effects

**Fig. 7:**
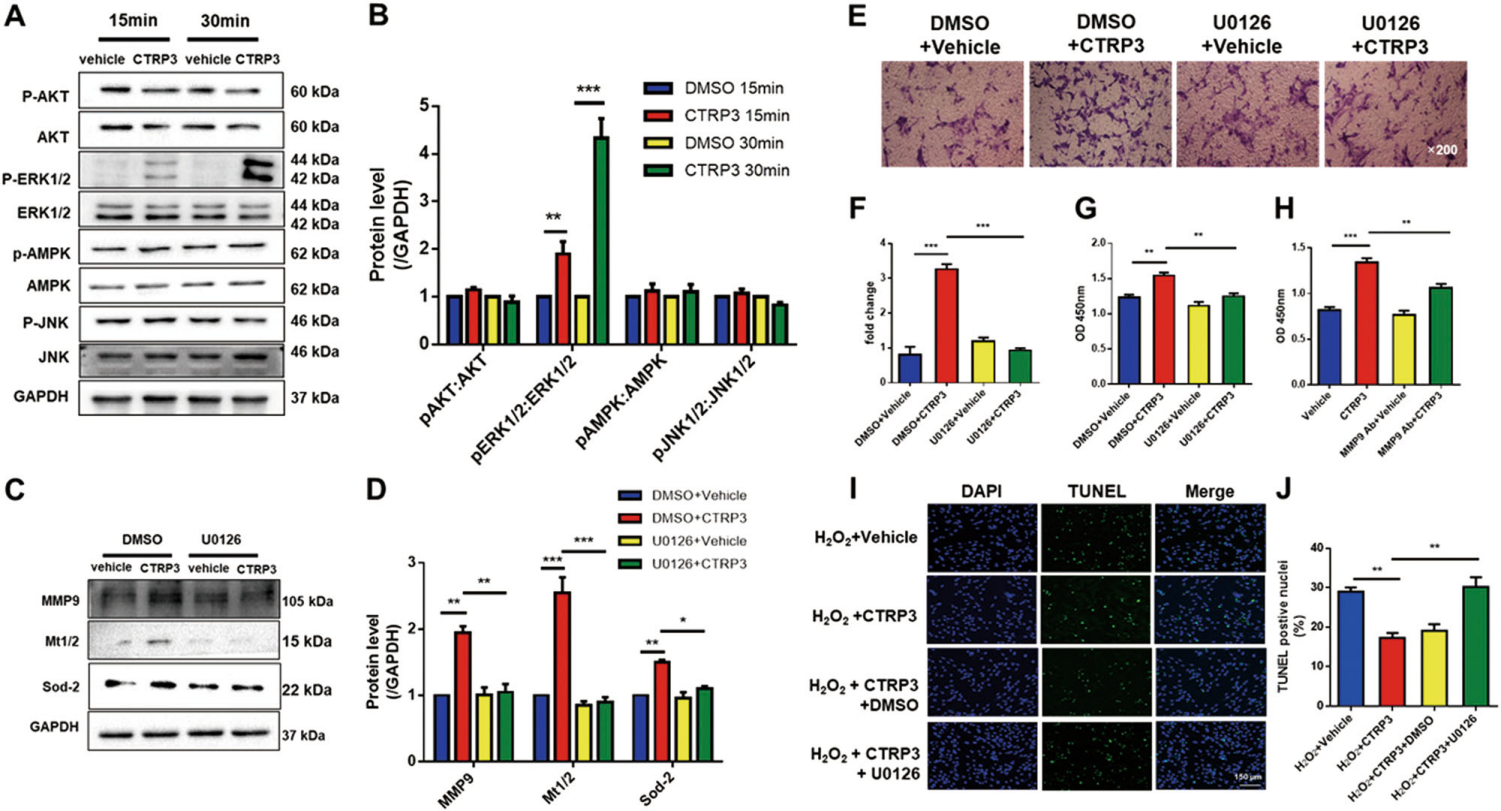
The ERK1/2 signaling pathway was responsible for the effects of CTRP3 on MSCs. **a**, **b** Western blot analysis of phospho-Akt/Akt, phospho-ERK1/2/ERK1/2, phospho-AMPK/AMPK, and phospho-JNK/JNK following CTRP3 (*n* = 3). **c**, **d** Effects of U0126 (an ERK1/2 inhibitor) on CTRP3-induced upregulation of MMP9, MT1/2, and SOD2. GAPDH served as a control (*n* = 4). **e**–**g** Effects of ERK1/2 inhibition on MSCs migration and proliferation (*n* = 4). Images were at original magnification, ×200. (H) CCK-8 assays were used to detect the effects of MMP9 neutralization on CTRP3-induced cell proliferation (*n* = 5). **i**, **j** Effects of ERK1/2 inhibition on apoptosis induced by oxidative stress via TUNEL staining (*n* = 4). All data are presented as means ± SEMs. *P < 0.05; **P < 0.01; ***P < 0.001

Among the screened upstream signaling molecules known to regulate cellular proliferation and survival by CTRP3^18,19^, ERK1/2 was identified as the most activated molecule. CTRP3 had no significant effect on AMPK, AKT, and JNK1/2 pathways (Fig. 7a, b). To determine whether ERK1/2 pathway was responsible for the CTRP3-mediated biological effects of MSCs, U0126 (which inhibited ERK1/2; 10 μM) was administered 2 h before hCTRP3 treatment, and U0126 completely blocked the upregulation of phosphorylated ERK1/2 induced by CTRP3 (Fig. S6). As shown in Fig. 7c, d, pretreatment with U0126 decreased CTRP3-induced MMP9 expression and completely blocked CTRP3-induced SOD2 and MT1/2 upregulation. Furthermore, by inhibiting the ERK1/2 pathway, MSCs migration (Fig. 7e, f), proliferation (Fig. 7g), and cytoprotective effects (Fig. 7i, j) induced by CTRP3 were abolished.

**Fig. 8:**
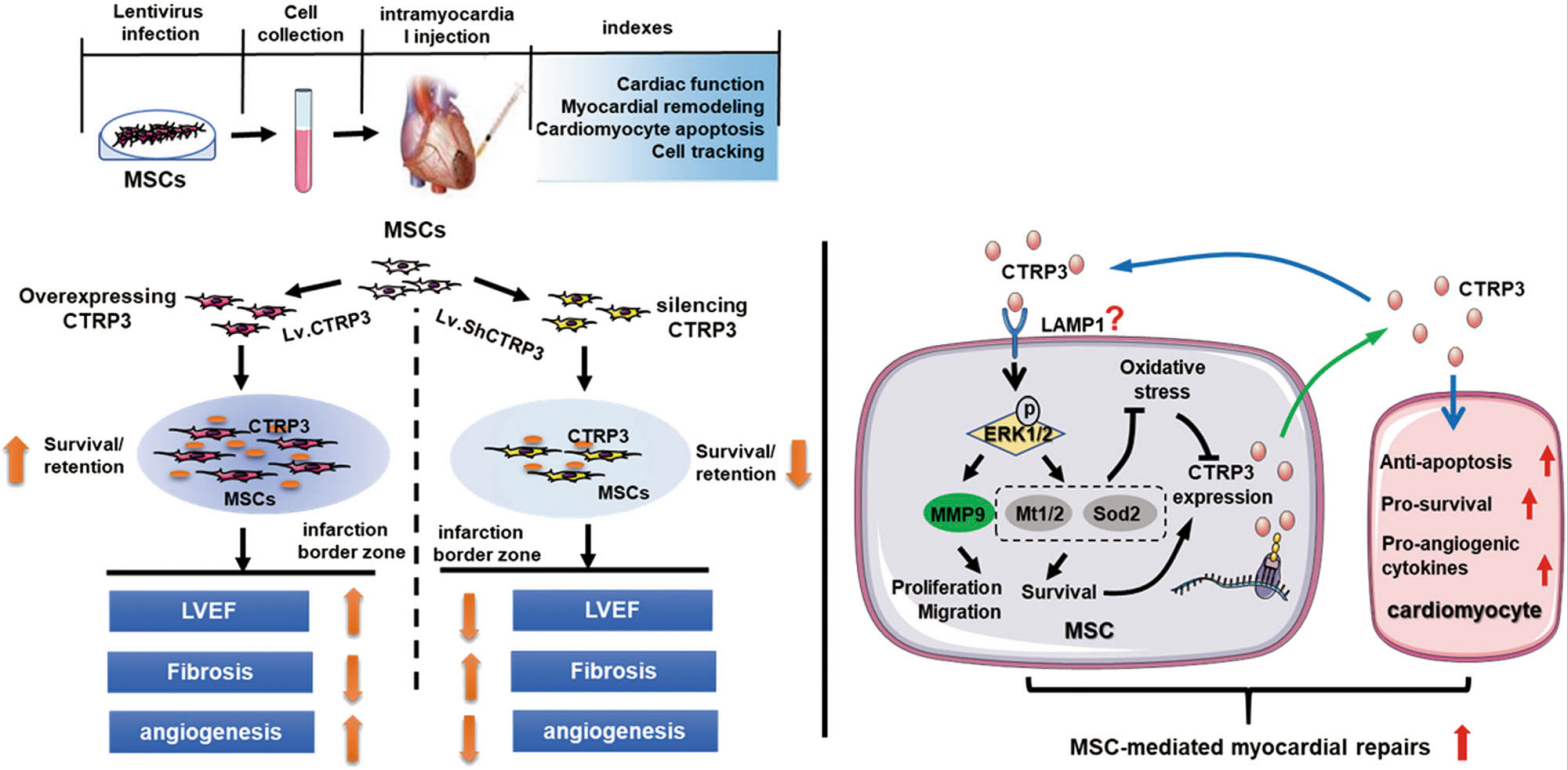
Schematic diagram of the enhanced effects of MSCs on myocardial repair induced by CTRP3 overexpression. Genetic manipulation of CTRP3 in MSCs restores CTRP3 levels. In vivo, overexpression of CTRP3 improves survival/retention of transplanted MSCs in ischemic microenvironment and enhances the therapeutic effect of MSCs on MI, however, knocking down CTRP3 impairs therapeutic efficacy of MSCs. Further, there is a positive feedback loop between MSCs and CTRP3. CTRP3 activates the ERK1/2-MMP9 pathway to promote proliferation and migration and the ERK1/2-Mt1/2/Sod2 pathway to improve survival, the latter of which resists oxidative stress and in turn upregulates CTRP3 expression. The released CTRP3 increases the retention/survival of MSCs in infarcted heart. In addition, CTRP3 inhibits myocardial apoptosis and induces the expression of the angiogenic factor VEGF as reported previously by us. CTRP3 overexpression in MSCs may be a novel therapeutic strategy that benefits both MSCs and cardiomyocytes and potentiates the curative effects of MSCs after MI

Finally, we investigated whether the CTRP3-ERK1/2-MMP9 axis functioned in the regulation of MSCs proliferation. To this end, an anti-MMP9 neutralizing antibody was administered together with CTRP3. As expected, blocking MMP9 repressed CTRP3-induced MSCs proliferation (Fig. 7h). These data suggested that CTRP3-ERK1/2-MMP9 pathway was involved in MSCs proliferation and migration, whereas CTRP3-ERK1/2-SOD2/MT1/2 pathway mediated MSCs cytoprotection. (Fig. 8)

## Discussion

In the current study, we first demonstrated that bone marrow MSCs expressed and secreted CTRP3 and oxidative simulation reduced CTRP3 expression and secretion in vitro. Moreover, CTRP3 overexpression in MSCs increased MSCs-mediated cardiac protection after MI by decreasing apoptosis, attenuating pathologic remodeling, and increasing vascularization and angiogenesis. Overexpression of CTRP3 potentiated MSCs survival and retention in the ischemia microenvironment. Additionally, knockdown of CTRP3 expression reduced MSCs survival/retention and attenuated MSCs efficacy on MI. We further found that MSCs secreted CTRP3 to enhance their proliferation, migration, and anti-apoptotic via activation of the ERK1/2 pathway and upregulation of MMP9 and MT1/2/SOD2.

Increasing evidence has demonstrated effectiveness and safety of MSCs in cardiovascular diseases in recent decades^20,21^. Although more work is needed to translate MSCs-based therapy to the clinical setting, this approach appears to be quite promising for the treatment of ischemic heart disease as shown in multiple clinical trials^20^. Current challenges are mostly low survival and retention of MSCs^2,22,23^. During hypoxic/ischemic injury, oxidative stress leads to myocyte loss and cardiac remodeling via multiple ways, including inducing cell apoptosis, and regulating growth factors signaling in the development of compensatory hypertrophy^24–26^. Oxidative stress has been also reported to lead to loss of transplanted MSCs^4^. Increased antioxidant capacity protect transplanted MSCs from the harsh microenvironment and improves MSCs efficacy for ischemic heart diseases^11,27^. Therefore, it is of great need to identify approaches to promote MSCs engraftment and survival.

Genetic modification has attracted great attention as an approach to overcome the low cellular retention^21^. Therefore, identifying suitable targets of MSCs for genetic modification and augmenting their survival is scientifically and clinically essential. CTRP3 is endogenously expressed in a variety of organisms and is highly conserved^18,28^. Although the protective role of direct CTRP3 administration in cardiovascular disease has been confirmed in previous studies^9,10^, it is unclear whether CTRP3-overexpressing MSCs exert beneficial effects on MI. Furthermore, the key roles of CTRP3 in protecting MSCs survival/retention under hypoxia/ischemia/oxidation stress remain unclear. In this study, we introduced a lentivirus expressing hCTRP3 into MSCs and injected infected MSCs into MI mice. The data showed that overexpression of CTRP3 significantly increased MSCs survival/retention in the ischemic microenvironment in vivo, thereby enhancing MSCs efficacy for MI. Moreover, silencing CTRP3 with shRNA impaired the therapeutic efficacy of MSCs owing to low survival/retention. These findings suggested that genetic modification of CTRP3 may serve as a promising alternative for protection of transplanted MSCs and augmentation of cell therapy.

Paracrine actions have been shown to contribute mainly to the therapeutic potential of MSCs by enhancing endogenous repair mechanisms after MI^21,29,30^. The *CTRP3* gene was first isolated in transforming growth factor-β1-treated mouse embryonic mesenchymal stromal cells and shown to be a secretory protein^31^. Later, Wong et al. identified CTRP3 as an adipokine classified within the CTRPs superfamily due to homologous molecular structures^32^. Recent reports have suggested that CTRP3 may have potential clinical applications. Circulating levels of CTRP3 decreased in patients with acute coronary syndrome^33^. Additionally, studies in animal models have confirmed that CTRP3 inhibits ventricular remodeling via induction of robust angiogenesis and reduction of interstitial fibrosis after MI^9,10^. In our study, we showed that CTRP3 was secreted by MSCs. Moreover, higher CTRP3 levels were found in the infarct border region after injection of CTRP3-overexpressing MSCs, implying that MSCs may provide improved drug delivery after MI than previously reported drug delivery vehicles (e.g., adenovirus or direct protein administration)^9–11,34^. Hence, the enhanced effects of CTRP3-overexpressing MSCs may be partly attributed to the paracrine signaling of CTRP3, although further studies are needed to confirm that. In addition, although delivery of MSCs after MI resulted in differentiation into vWF-positive cells, the percentage of differentiated cells was very low, and CTRP3 failed to regulate MSCs trans-differentiation, indicating that multilineage differentiation may not be the main mechanisms, consistent with previous reports^35,36^.

Previous reports have shown that MSCs regulate their own biological behaviors via autocrine mechanisms^37–39^. Our work in vitro provided an autocrine explanation for the cellular and molecular mechanisms responsible for increased MSCs survival/retention in vivo by CTRP3. In this study, we demonstrated that MSCs migration was higher when cells were cultured with LvC3-MSCs than with LvNull-MSCs. Furthermore, the effects were inhibited when cells were cocultured with C3^−/−^ MSCs. Moreover, CTRP3-enriched CM protected MSCs against oxidative stress-induced cell apoptosis/death, which was compromised when MSCs were incubated with non-CTRP3 CM. Moreover, CTRP3 protein alone mimicked the above-mentioned effects. Taken together, these findings suggested that there were unknown receptors mediating these functions. lysosomal-associated membrane protein 1 (LAMP1) is expressed widely in multiple cell types and regarded as a marker for endosomes and lysosomes^40^. LAMP1 is observed on the plasma membrane, indicating that it may serve as a cell surface receptor^41^. Furthermore, recent reports have identified LAMP1 as a potential receptor for CTRP3^42^. In addition, LAMP1 has been shown to mediate ERK1/2 activity, leading to MSCs proliferation^43^. Our data also showed that CTRP3 increased MSCs proliferation via ERK1/2 pathway, suggesting that LAMP1 may mediate the autocrine effects of CTRP3 in MSCs; however, further studies are needed to support the hypothesis. With respect to molecular mechanisms, several kinases have been shown to regulate the effects of CTRP3 on cell proliferation, migration, and survival^19^, and only ERK1/2 is activated by CTRP3 in MSCs. Furthermore, we identified two pathways, namely, the CTRP3-ERK1/2-MMP9 axis for proliferation and promigration effects and the CTRP3-ERK1/2-MT1/2/SOD2 axis for anti-apoptotic and prosurvival effects. Therefore, our results implied that CTRP3 may serve as an autocrine factor modulating MSCs functions.

In summary, our findings identified CTRP3 as a possible autocrine modulator of the proliferation, migration, and survival of MSCs. Importantly, overexpression of CTRP3 enhanced MSCs survival/retention in vivo and improved MSCs efficacy for myocardial repair. Furthermore, our current results, combined with our previous report showing that CTRP3 supplementation induced angiogenesis and attenuated post-MI remodeling, suggested that locally enriched CTRP3 may function as a regulatory factor within the cardiac niche, both contributing to the survival of implanted cells and protecting ischemic myocardium.

## Supplementary information

Supplementary meterials
