# Supplementary material for: C1q/tumor necrosis factor-related protein-3-engineered mesenchymal stromal cells attenuate cardiac impairment in mice with myocardial infarction
Source: Cell Death Dis. 2019 Jul 11;10(7):530. doi: 10.1038/s41419-019-1760-5 (PMC6624206; doi:10.1038/s41419-019-1760-5)
Supplement: Supplementary file 1 — Supplementary meterials [file 41419_2019_1760_MOESM1_ESM.docx]

**Table S1**

**Real Time PCR Primers**

| **Primer name** | **Sequence (5'to 3')** | **Primer name** | **Sequence (5'to 3')** |
| --- | --- | --- | --- |
| Gapdh--F | AGGTCGGTGTGAACGGATTTG | Tgfb2--F | TCGACATGGATCAGTTTATGCG |
| Gapdh--R | TGTAGACCATGTAGTTGAGGTCA | Tgfb2--R | CCCTGGTACTGTTGTAGATGGA |
| Actb (β-actin)--F | GGCTGTATTCCCCTCCATCG | Mt1--F | AAGAGTGAGTTGGGACACCTT |
| Actb (β-actin)--R | CCAGTTGGTAACAATGCCATGT | Mt1--R | CGAGACAATACAATGGCCTCC |
| Ccna2--F | GCCTTCACCATTCATGTGGAT | Mt2--F | GCCTGCAAATGCAAACAATGC |
| Ccna2--R | TTGCTGCGGGTAAAGAGACAG | Mt2--R | AGCTGCACTTGTCGGAAGC |
| Ccnb1--F | AAGGTGCCTGTGTGTGAACC | Mt3--F | ACCTGCCCCTGTCCTACTG |
| Ccnb1--R | GTCAGCCCCATCATCTGCG | Mt3--R | CCTTGGCACACTTCTCACATC |
| Ccnd1--F | GCGTACCCTGACACCAATCTC | Sod1--F | AACCAGTTGTGTTGTCAGGAC |
| Ccnd1--R | CTCCTCTTCGCACTTCTGCTC | Sod1--R | CCACCATGTTTCTTAGAGTGAGG |
| Ccne1--F | GTGGCTCCGACCTTTCAGTC | Sod2--F | CAGACCTGCCTTACGACTATGG |
| Ccne1--R | CACAGTCTTGTCAATCTTGGCA | Sod2--R | CTCGGTGGCGTTGAGATTGTT |
| Cdkn2a (p16)--F | CGCAGGTTCTTGGTCACTGT | Sod3--F | CCTTCTTGTTCTACGGCTTGC |
| Cdkn2a (p16)--R | TGTTCACGAAAGCCAGAGCG | Sod3--R | TCGCCTATCTTCTCAACCAGG |
| Cdkn1a (p21)--F | CCTGGTGATGTCCGACCTG | Nrf1--F | AGCACGGAGTGACCCAAAC |
| Cdkn1a (p21)--R | CCATGAGCGCATCGCAATC | Nrf1--R | TGTACGTGGCTACATGGACCT |
| Trp53 (p53)--F | GCGTAAACGCTTCGAGATGTT | Nfe2l2--F | TCTTGGAGTAAGTCGAGAAGTGT |
| Trp53 (p53)--R | TTTTTATGGCGGGAAGTAGACTG | Nfe2l2--R | GTTGAAACTGAGCGAAAAAGGC |
| Pak3--F | TTGGATAACGAAGAAAAACCCCC | Txn2--F | TGGGCTTCCCTCACCTCTAAG |
| Pak3--R | GAGTCTCGGTTGTTACTGTTCAT | Txn2--R | CCTGGACGTTAAAGGTCGTCA |
| Racgap1--F | CGCCGGATGGAGATTATCAATG | Hmox1--F | AAGCCGAGAATGCTGAGTTCA |
| Racgap1--R | CCCCGTCTCTGCTTTCAACAA | Hmox1--R | GCCGTGTAGATATGGTACAAGGA |
| Nusap1--F | CGTCACCAAAACGAGGAGGAG | Nqo1--F | AGGATGGGAGGTACTCGAATC |
| Nusap1--R | AGAAAACTCATCCGTGCATAGAG | Nqo1--R | AGGCGTCCTTCCTTATATGCTA |
| Myh10--F | GGAATCCTTTGGAAATGCGAAGA | Gpx1--F | AATGTCGCGTCTCTCTGAGG |
| Myh10--R | GCCCCAACAATATAGCCAGTTAC | Gpx1--R | TCCGAACTGATTGCACGGG |
| Cks2--F | TCGATGAGCACTACGAGTACC | Gpx4--F | GATGGAGCCCATTCCTGAACC |
| Cks2--R | CCATCCTAGACTCTGTTGGACAC | Gpx4--R | CCCTGTACTTATCCAGGCAGA |
| Mmp2--F | CAAGTTCCCCGGCGATGTC | Prdx1--F | AATGCAAAAATTGGGTATCCTGC |
| Mmp2--R | TTCTGGTCAAGGTCACCTGTC | Prdx1--R | CGTGGGACACACAAAAGTAAAGT |
| Mmp3--F | ACATGGAGACTTTGTCCCTTTTG | Ndufb5--F | CAAGAGACTGTTTGTCGTCAAGC |
| Mmp3--R | TTGGCTGAGTGGTAGAGTCCC | Ndufb5--R | TGTTCACCAGTGTTATGCCAAT |
| Mmp9--F | GCAGAGGCATACTTGTACCG | Sdha--F | GGAACACTCCAAAAACAGACCT |
| Mmp9--R | TGATGTTATGATGGTCCCACTTG | Sdha--R | CCACCACTGGGTATTGAGTAGAA |
| Mmp12--F | GAGTCCAGCCACCAACATTAC | Uqcrc2--F | AAAGTTGCCCCGAAGGTTAAA |
| Mmp12--F | GCGAAGTGGGTCAAAGACAG | Uqcrc2--R | GAGCATAGTTTTCCAGAGAAGCA |
| Mmp19--F | CTGTGGCTGGCATTCTTACTT | Cox6a1--F | TCAACGTGTTCCTCAAGTCGC |
| Mmp19--R | GGGCAGTCCAGATGCTTCC | Cox6a1--R | AGGGTATGGTTACCGTCTCCC |
| Bcl2--F | GTCGCTACCGTCGTGACTTC | Atp5b--F | GGTTCATCCTGCCAGAGACTA |
| Bcl2--R | CAGACATGCACCTACCCAGC | Atp5b--R | AATCCCTCATCGAACTGGACG |
| Bcl2l1--F | GACAAGGAGATGCAGGTATTGG | Mdh2--F | TTGGGCAACCCCTTTCACTC |
| Bcl2l1--R | TCCCGTAGAGATCCACAAAAGT | Mdh2--R | GCCTTTCACATTTGCTCTGGTC |
| Dapk1--F | ATGACTGTGTTCAGGCAGGAA | Idh2--F | GGAGAAGCCGGTAGTGGAGAT |
| Dapk1--R | CCGGTACTTTTCTCACGACATTT | Idh2--R | GGTCTGGTCACGGTTTGGAA |
| Stk17b--F | ATGTCTCGGAGGAGATTCGATT | Pou5f1 (OCT4)--F | GGCTTCAGACTTCGCCTCC |
| Stk17b--R | TTTTCAGGGATTTGGCAGCAT | Pou5f1 (OCT4)--R | AACCTGAGGTCCACAGTATGC |
| Becn1--F | ATGGAGGGGTCTAAGGCGTC | Nanog--F | TCTTCCTGGTCCCCACAGTTT |
| Becn1--R | TCCTCTCCTGAGTTAGCCTCT | Nanog--R | GCAAGAATAGTTCTCGGGATGAA |
| Map1lc3a (LC3)--F | TTATAGAGCGATACAAGGGGGAG | Nkx2-5--F | TGACCCAGCCAAAGACCCT |
| Map1lc3a (LC3)--R | CGCCGTCTGATTATCTTGATGAG | Nkx2-5--R | CCATCCGTCTCGGCTTTGT |
| Sqstm1 (p62)--F | ATGTGGAACATGGAGGGAAGA | Kit (c-KIT)--F | CTCCCCCAACAGTGTATTCAC |
| Sqstm1 (p62)--R | GGAGTTCACCTGTAGATGGGT | Kit (c-KIT)--R | TAGCCCGAAATCGCAAATCTT |
| Kitl (SCF)--F | CGGGATGGATGTTTTGCCTA | Nppa--F | GCTTCCAGGCCATATTGGAG |
| Kitl (SCF)--R | CTTCGGTGCGTTTTCTTCCA | Nppa--R | GGGGGCATGACCTCATCTT |
| Cxcl12 (Sdf1)--F | TGCATCAGTGACGGTAAACCA | Gata4--F | CCCTACCCAGCCTACATGG |
| Cxcl12 (Sdf1)--R | CACAGTTTGGAGTGTTGAGGAT | Gata4--R | ACATATCGAGATTGGGGTGTCT |
| Fgf2--F | GCTGCTGGCTTCTAAGTGTG | Pecam1 (CD31)--F | CTGCCAGTCCGAAAATGGAAC |
| Fgf2--R | TACTGCCCAGTTCGTTTCAG | Pecam1 (CD31)--R | CTTCATCCACCGGGGCTATC |
| Hgf--F | ATGTGGGGGACCAAACTTCTG | Kdr (Flk-1)--F | TTTGGCAAATACAACCCTTCAGA |
| Hgf--R | GGATGGCGACATGAAGCAG | Kdr (Flk-1)--R | GCAGAAGATACTGTCACCACC |
| Igf1--F | CTGGACCAGAGACCCTTTGC | Cdh5 (VEcad)--F | CACTGCTTTGGGAGCCTTC |
| Igf1--R | GGACGGGGACTTCTGAGTCTT | Cdh5 (VEcad)--R | GGGGCAGCGATTCATTTTTCT |
| Pdgfra--F | AGGTATGTATCCACACATGCGT | Cldn5 (Claudin 5)--F | GCAAGGTGTATGAATCTGTGCT |
| Pdgfra--R | AGTTCCTGTTGGTTTCATCTCG | Cldn5 (Claudin 5)--R | GTCAAGGTAACAAAGAGTGCCA |
| Pdgfrb--F | TTCCAGGAGTGATACCAGCTT | Tagln (Sm22a)--F | CAACAAGGGTCCATCCTACGG |
| Pdgfrb--R | AGGGGGCGTGATGACTAGG | Tagln (Sm22a)--R | ATCTGGGCGGCCTACATCA |
| Angpt1--F | CACATAGGGTGCAGCAACCA | Acta2 (a-SMA)--F | GTCCCAGACATCAGGGAGTAA |
| Angpt1--R | CGTCGTGTTCTGGAAGAATGA | Acta2 (a-SMA)--R | TCGGATACTTCAGCGTCAGGA |
| Angptl2--F | AGCCTGAGAATACCAACCGC | Cnn1 (Calponin 1)--F | TCTGCACATTTTAACCGAGGTC |
| Angptl2--R | CCCTTGCTTATAGGTCTCCCAG | Cnn1 (Calponin 1)--R | GCCAGCTTGTTCTTTACTTCAGC |
| Vegfa--F | GCACATAGAGAGAATGAGCTTCC | Myh11 (smMHC)--F | AAGCTGCGGCTAGAGGTCA |
| Vegfa--R | CTCCGCTCTGAACAAGGCT | Myh11 (smMHC)--R | CCCTCCCTTTGATGGCTGAG |

**Supplemental Figures and Figure Legends**


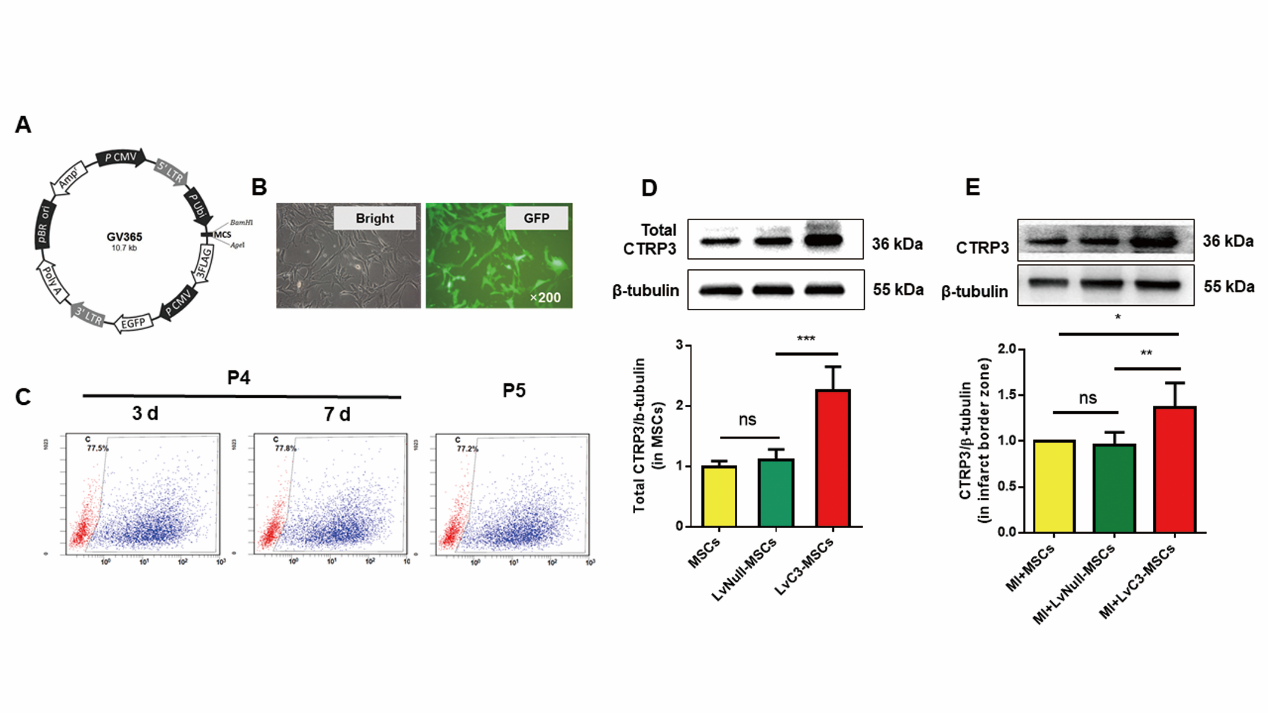


**Figure S1: Construction of a lentiviral vector overexpressing hCTRP3**

(A) Vector information about GV 365 (Ubi-MCS-3FLAG-CMV-EGFP). (B) Representative image of MSC morphology (passage 4) before (bright) and after transduction (GFP). Images were at original magnification, X200. (C) P4 MSCs were infected and infection efficiency was assessed by flow cytometry at 3 days and 7 days post-infection. P5 MSCs were obtained from cell passage of P4 MSCs at 7 days post-infection and the proportion of GFP positive cells in P5 MSCs was measured by flow cytometry. (D) Western blots and quantification of CTRP3 protein expression in infected MSCs (n = 6). (E) Western blots and quantification of CTRP3 protein expression in the infarct border zone 7 days after MSC transplantation (n = 5). Data are means ± SEMs. **P* < 0.05, ***P* < 0.01, ****P* < 0.001. ns, not significant. Data are means ± SEMs.


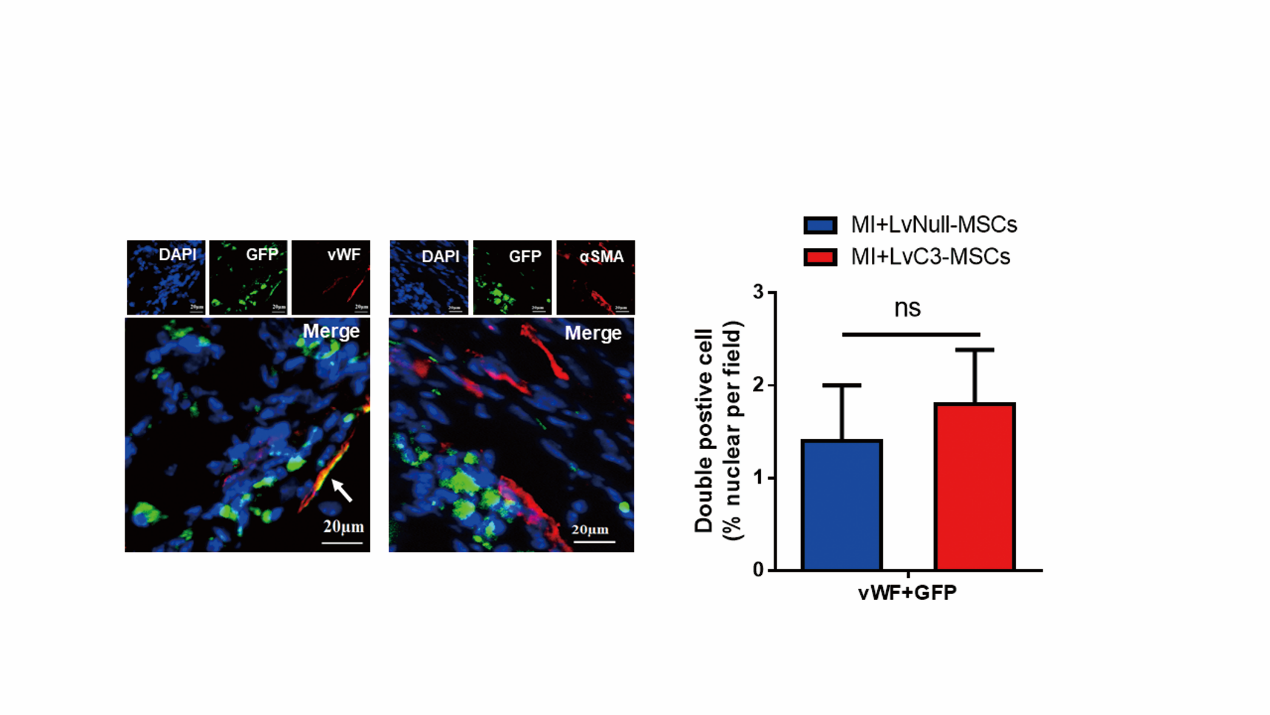


**Figure S2: CTRP3 overexpression rarely contributed to MSC differentiation in vivo**

Representative confocal microscopic images of vWF (red) and αSMA（red）in the infarct region 7 days after MSC administration. Injected MSCs were positive for green fluorescent protein (green). Nuclei were stained with DAPI (blue; n = 5). White arrows show representative MSC-derived ECs. MSC-derived SMCs were not found. The bar graph shows the cell density. All data are expressed as means ± SEMs. ns, not significant.


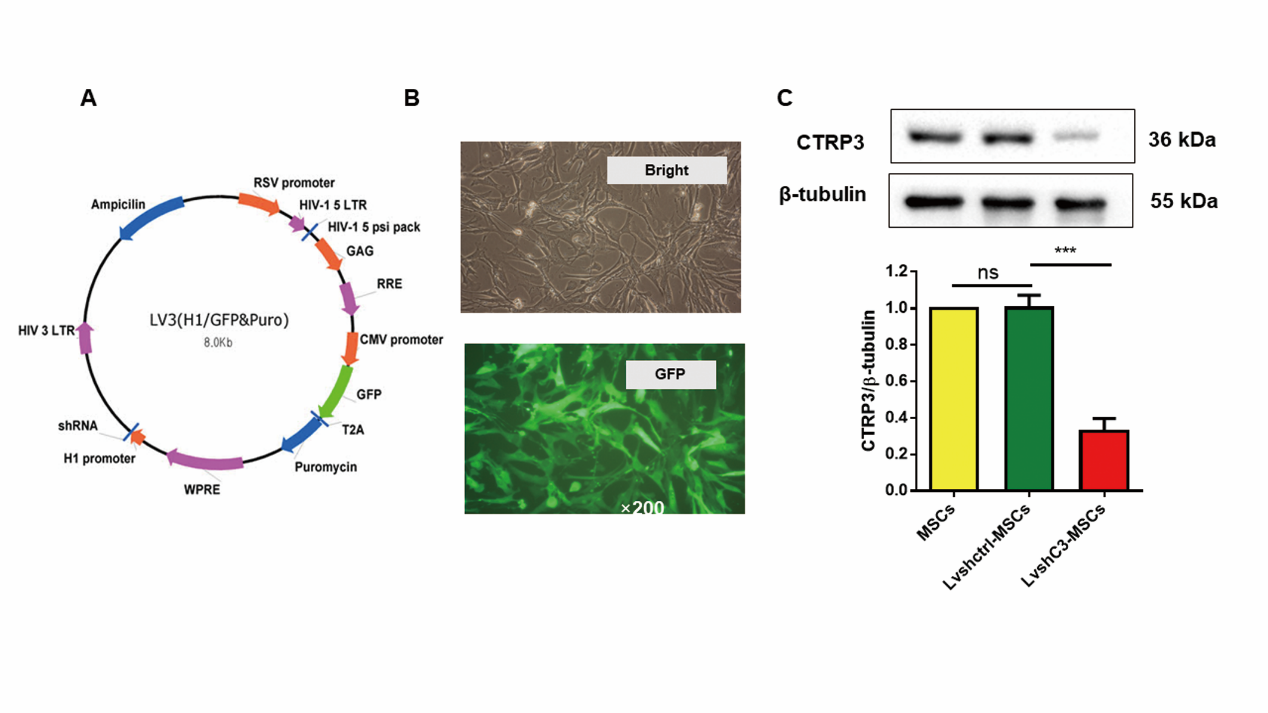


**Figure S3: Infection of MSCs with LvshC3**

(A) The map of shRNA lentiviral vector LV-3. (B) Representative image of MSCs after LvshC3 and Lvshctrl transduction. Images were at original magnification, X200. (C) Western blotting was used to detect CTRP3 expression in LvshC3-MSCs (n = 6). All data are presented as means ± SEMs. ****P* < 0.001. ns, not significant.


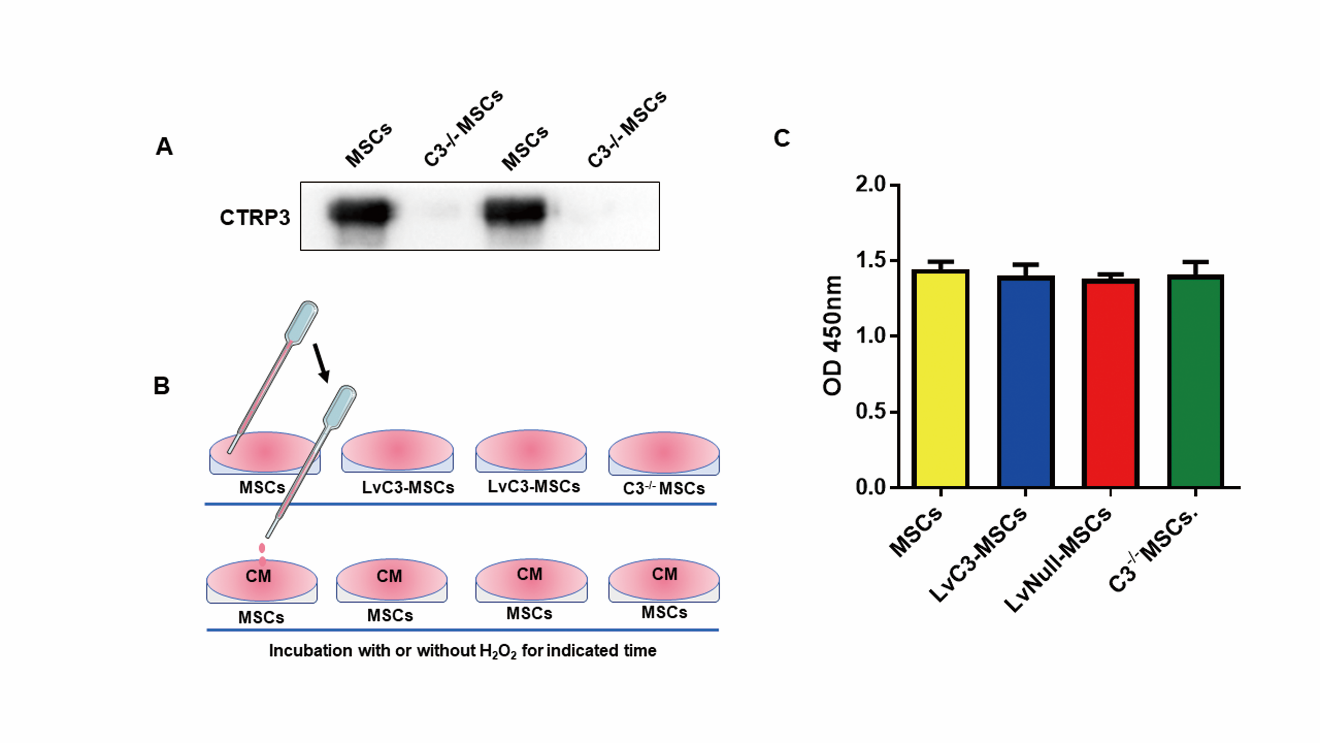


**Figure S4: neither CTRP3-enriched CM nor CTRP3-deficient CM had pronounced effects on MSC proliferation**

(A) Detection of CTRP3 expression in CM from C3^-/-^ MSCs by western blotting. (B) Experimental setting detecting effects of CTRP3 secreted by MSCs on MSC proliferation and oxidation resistance are illustrated in the schematic diagram. (C) Proliferation of MSCs after incubation with different CMs, as measured by CCK-8 assays (n = 6). Data are means ± SEMs. ns, not significant.


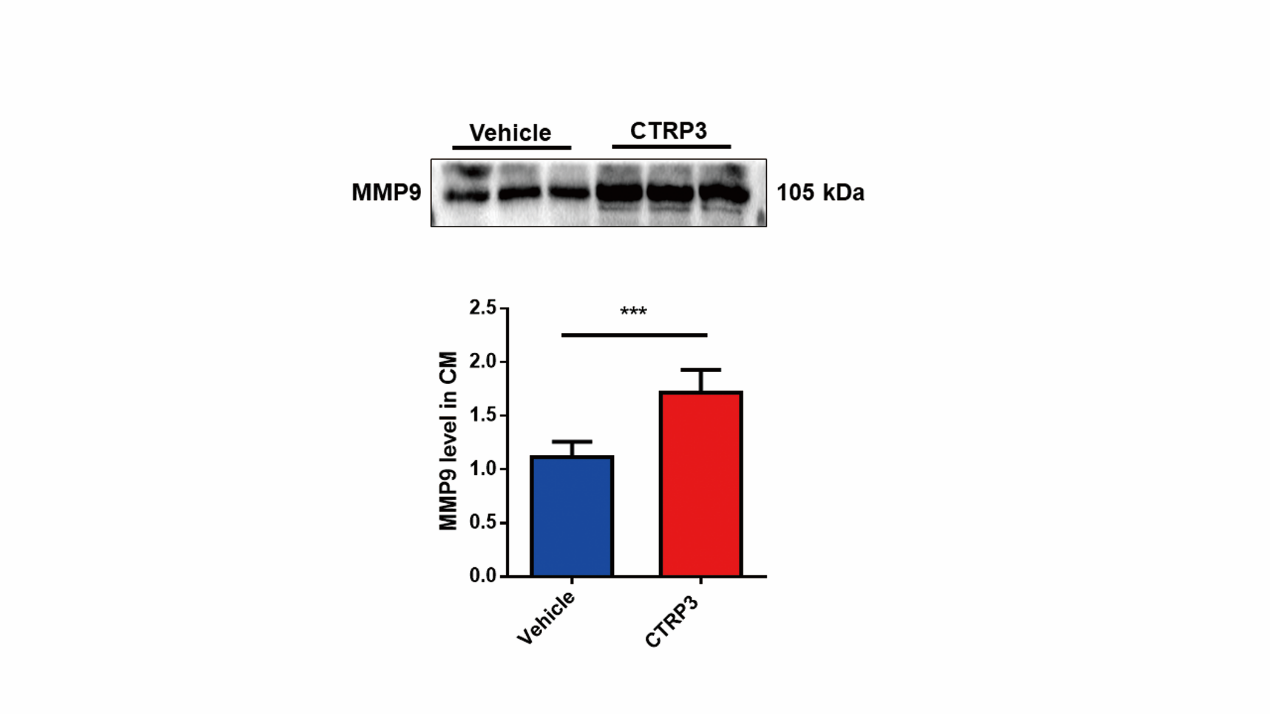


**Figure S5: CTRP3 upregulated MMP9 secretion in MSCs**

Western blotting and quantification of MMP-9 levels in CM from MSCs (n = 6). Data are means ± SEMs. ****P* < 0.001.


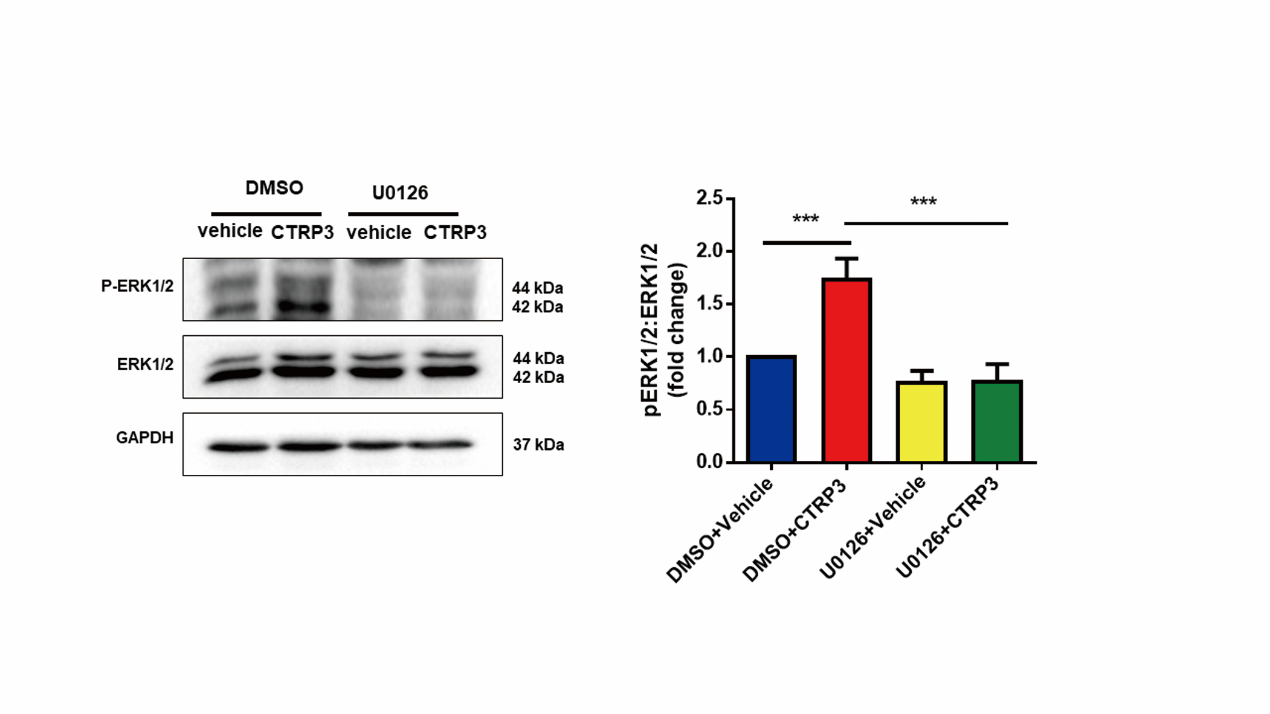


**Figure S6: U0126 significantly inhibited ERK1/2 activation by CTRP3 in MSCs.**

(A) Western blotting and (B) quantitative analysis (n = 5). Data are means ± SEMs. ****P* < 0.001.
